# Impact of Single-Nucleotide Polymorphisms of *CTLA-4*, *CD80* and *CD86* on the Effectiveness of Abatacept in Patients with Rheumatoid Arthritis

**DOI:** 10.3390/jpm10040220

**Published:** 2020-11-11

**Authors:** Noelia Marquez Pete, María del Mar Maldonado Montoro, Cristina Pérez Ramírez, Almudena Sánchez Martín, Juan Enrique Martínez de la Plata, Fernando Martínez Martínez, Rafael Caliz Caliz, Abdelali Daddaoua, María del Carmen Ramírez Tortosa, Alberto Jiménez Morales

**Affiliations:** 1 Pharmacy Service. Pharmacogenetics Unit, University Hospital Virgen de las Nieves, Avda. Fuerzas Armadas, 2, 18014 Granada, Spain; noeliamarquezpete@gmail.com (N.M.P.); almuweb06@gmail.com (A.S.M.); alberto.jimenez.morales.sspa@juntadeandalucia.es (A.J.M.); 2 Clinical Analysis Service, Hospital Campus de la Salud, Av. de la Investigación, 18016 Granada, Spain; mariadelmarmaldonadomontoro@gmail.com; 3 Pharmacy Service. Pharmacogenetics Unit, University Hospital Virgen Macarena, Dr. Fedriani, 3, 41009 Sevilla, Spain; 4 Pharmacy Service. Hospital de Poniente-El Ejido, Diseminado Ctra Almerimar, 31, 04700 El Ejido, Spain; juanenriquemartinezdelaplata@gmail.com; 5 Department of Physical Chemistry, Faculty of Pharmacy, University of Granada, Campus Universitario de Cartuja, 18071 Granada, Spain; femartin@ugr.es; 6 Department of Pharmacy and Pharmaceutical Technology. Social and Legal Assistance Pharmacy Section, Faculty of Pharmacy, University of Granada, Campus Universitario de Cartuja, 18071 Granada, Spain; 7 Rheumatology Service, University Hospital Virgen de las Nieves, Avda. Fuerzas Armadas, 2, 18014 Granada, Spain; rcalizcaliz@gmail.com; 8 Department of Biochemistry, Faculty of Pharmacy, University of Granada, Campus Universitario de Cartuja, 18071 Granada, Spain; daddaoua@ugr.es (A.D.); mramirez@ugr.es (M.d.C.R.T.)

**Keywords:** rheumatoid arthritis, abatacept, CTLA4, effectiveness, polymorphisms

## Abstract

Abatacept (ABA) is used as a first-line treatment in patients diagnosed with moderate and severe rheumatoid arthritis (RA). The interindividual response to ABA therapy is very variable in these patients. The objective of our study was therefore to investigate the role of polymorphisms of the *CTLA-4*, *CD80* and *CD86* genes, as well as that of clinical factors of the disease, in the response to ABA in patients with RA. A retrospective cohort study was carried out in 109 patients receiving treatment with ABA and diagnosed with RA. The genetic variables were analyzed using real-time PCR with TaqMan^®^ probes. The patients were classified according to the European League Against Rheumatism (EULAR) criteria at 6 and 12 months from start of treatment. The independent variables associated with higher EULAR response were lower duration of previous biologic disease-modifying anti-rheumatic drugs and lower baseline values of the disease activity score 28 after 6 months of ABA treatment; and lower baseline patient’s visual analogue scale (PVAS) after 12 months. In addition, a significant association was found between duration of ABA treatment, non-administration of concomitant glucocorticoids and lower baseline values of the number of inflamed joints and erythrocyte sedimentation rate clinical variables, with remission of the disease after 6 months’ treatment with ABA. Finally, remission of the disease after 12 months’ treatment with ABA was associated with earlier age at start of ABA therapy and lower number of previous biologic therapies (BTs). The *CTLA-4* *rs5742909-T* allele and the *CTLA-4* *rs231775-G* allele were found to be associated with satisfactory EULAR response and low disease activity (LDA) after 12 months’ treatment with ABA (*CTLA-4* *rs5742909 T *vs.* CC*; OR = 5.88; CI_95%_ = 1.48–23.29 and OR = 4.75; CI_95%_ = 1.35–17.94, respectively, and *CTLA-4* *rs231775 G *vs.* AA*, OR = 3.48; CI_95%_ = 1.20–10.09 and OR = 4.68; CI_95%_ = 1.49–17.94, respectively). In conclusion, patients with RA treated with ABA showed better EULAR response and LDA rate when they had the *CTLA-4 rs5742909-T* or *CTLA-4 rs231775-G* polymorphisms; furthermore, this remission rate increased in patients that began ABA treatment earlier, those with a lower number of previous BTs and those with a lower PVAS value.

## 1. Introduction

Rheumatoid arthritis (RA) is a chronic autoimmune disease affecting the joints. It produces inflammation and structural damage, reducing patients’ quality of life [1]. The worldwide prevalence of the disease varies between 0.3% and 1.2%, and it is the most common chronic inflammatory joint pathology in Spain, with a prevalence of between 0.3% and 1.6% [2,3,4,5]. The therapeutic strategy for RA has changed in recent years. Clinical remission or, failing that, low disease activity (LDA) has been established as the main objective [6]. Biologic disease-modifying antirheumatic drugs (bDMARDs), as well as targeted synthetic DMARDs (tsDMARDs), have played a key role in the prognosis of RA [7]. One of the therapeutic targets indicated in RA treatment is abatacept (ABA). This is a fusion protein formed by the extracellular domain of cytotoxic T-lymphocyte-associated antigen 4 (CTLA-4), linked to the constant fragment (Fc) of human immunoglobulin 1 (IgG1). The mechanism of action of ABA is based on its binding with the CD80/CD86 complex, preventing the interaction of this complex with the CD28 transmembrane protein of T-lymphocytes by blocking the co-stimulation signal necessary for their activation [8].

Abatacept is used as a first-line treatment for moderate and severe RA in patients in whom the therapeutic objective has not been achieved after previous administration of DMARDs, according to the criteria of the European League Against Rheumatism (EULAR) recommendations [7]. There is great interindividual variability in treatment response with bDMARDs, with a therapeutic failure rate of approximately 30% [9]. Finding response biomarkers for these drugs would help to develop a therapy individually tailored to the patient, achieving the therapeutic objective more quickly and effectively. Interindividual genetic variability may also contribute to the response to ABA treatment. Single-nucleotide polymorphisms (SNPs) have been associated with response to bDMARDs and could be used as pharmacogenetic predictors [10,11,12,13,14].

Polymorphisms in the *CD80* and *CD86* genes have previously been studied for their association with autoimmune diseases [15,16,17,18]. The CD80/CD86 complex is a set of membrane glycoproteins consisting of two extracellular domains: a transmembrane domain and a cytoplasmic tail domain [19,20]. In the *CD86* gene we find the *rs1129055* (*G* > *A*) polymorphism, located in exon 8, which gives rise to an alanine (Ala) to threonine (Thr) amino acid substitution at codon 304 [21]. Similarly, in the *CD80* gene we find the *rs57271503* (*G* > *A*) polymorphism. Furthermore, SNPs in the *CTLA-4* gene have been associated with peripheral tolerance. CTLA-4 is an immune system regulatory receptor, constituently expressed on the surface of activated T lymphocytes and in regulatory T lymphocytes, inhibiting their proliferation [22,23,24]. CTLA-4 shows a greater affinity for the CD80/CD86 complex than CD28 and activates an immune signal inhibiting T-lymphocyte proliferation. It is indispensable for terminating the immune response cascade, as well as for acquisition of peripheral tolerance for antigens, preventing autoimmunity [23,25].

All these receptors are related, in turn, to the mechanism of action of ABA [15,18,26,27,28]. The presence of various SNPs in these genes may entail a conformational change in the proteins they encode, producing variability in the patient’s treatment response. In this conceptual framework, our objective is to assess the role of SNPs of these three genes, *CTLA-4* (*rs3087243*, *rs231775* and *rs5742909*), *CD80* (*rs57271503*) and *CD86* (*rs1129055*), as possible response predictors (EULAR response, LDA and remission) in patients with RA treated with ABA.

## 2. Material and Methods

### 2.1. Study Design

We conducted a retrospective cohort study.

### 2.2. Ethics Statements

This study was carried out in accordance with the Declaration of Helsinki, with the approval of the Ethics and Research Committee of the University Hospital Virgen de las Nieves. The subjects who participated in the study signed an informed consent for collection and genetic analysis of saliva samples and for their donation to the Andalusian Public Health System Biobank. The samples were identified by alphanumeric codes.

### 2.3. Study Population

The study included 109 Caucasian patients diagnosed with RA according to the American College of Rheumatology (ACR) classification criteria, recruited in the Rheumatology and Pharmacy Departments of the University Hospital Virgen de las Nieves in Granada between 2009 and 2019 [7]. Of the 109 patients recruited in the study, the response to ABA was evaluated in 105 after 6 months and in 92 after 12 months. The remaining patients did not meet the study’s evaluation criteria. The participants were over the age of 18 years and being treated with ABA. The route of administration of the drug was intravenous (IV): 500 mg (<60 kg), 750 mg (60–100 kg) or 1000 mg (>100 kg), in weeks 0, 2 and 4, respectively, and subsequently every 4 weeks at the same dose, in 49 patients; and subcutaneous (SC): 125 mg/week, in 60 patients.

### 2.4. Sociodemographic and Clinical Variables

The sociodemographic variables included sex, smoking, age at diagnosis of RA, number of years with the disease, age at initiation and duration of ABA treatment, route of administration of the drug (IV or SC), concomitant glucocorticoids (GCs), concomitant conventional synthetic DMARDs (csDMARDs) (methotrexate (MTX), leflunomide (LFN)), number and duration of previous BTs and reason for suspension of ABA.

The clinical variables collected were Disease Activity Score 28 (DAS28) [29,30,31], Health Assessment Questionnaire (HAQ) score, C-reactive protein (CRP) level, erythrocyte sedimentation rate (ESR), presence of rheumatoid factor (RF), anti-cyclic citrullinated peptide antibodies (ACPAs), number of painful joints (NPJ), number of inflamed joints (NIJ) and patient’s visual analogue scale (PVAS).

### 2.5. Genetic Variables

#### 2.5.1. DNA Isolation

The saliva samples were collected with buccal swabs (Kit OCR-100). The DNA was extracted using the QlAamp DNA Mini Kit (Qiagen GmbH, Hilden, Germany), following the manufacturer’s instructions for purifying DNA from saliva, and stored at −40 °C. The DNA concentration and purity were measured using a NanoDrop 2000 UV spectrophotometer with the absorbance ratio at 280/260 and 280/230.

#### 2.5.2. Detection of Gene Polymorphisms

The *CTLA-4 rs3087243*, *CTLA-4 rs231775*, *CTLA-4 rs5742909*, *CD80 rs57271503* and *CD86 rs1129055* gene polymorphisms were analyzed by real-time polymerase chain reaction (PCR) for allelic discrimination using TaqMan^®^ probes (ABI Applied Biosystems, 7300 Real-Time PCR System), following the manufacturer’s instructions. The assay ID for *CTLA-4 rs3087243* is C___3296043_10, for *CTLA-4 rs231775* is C___2415786_20, for *CTLA-4 rs5742909* is C__27834180_10, for *CD80 rs57271503* is C____387937_10 and for *CD86 rs1129055* is C___7504226_10. The genotypic determination was performed by StepOne v2.3 software.

### 2.6. Response Variables

The effectiveness of the treatment was evaluated according to the EULAR response, LDA and disease remission criteria, at 6 and 12 months from commencement of treatment with ABA.

The EULAR response was evaluated according to the European League Against Rheumatism criteria and classified as satisfactory (DAS28 < 3.2) or unsatisfactory (DAS28 ≥ 3.2) [7,32,33]. The LDA was established for values in the range 2.6 ≥ DAS28 ≥ 3.2 [31,33] and remission for DAS28 < 2.6 [7,33,34].

### 2.7. Statistical Analysis

The descriptive analysis was performed using R 3.5.1 software. The quantitative variables were expressed as the mean (± standard deviation) for those that complied with normality and as the median and percentiles (25 and 75) for the variables that did not follow a normal distribution. Normality was confirmed by the Shapiro–Wilk test.

The bivariate analysis between the response and the sociodemographic, clinical and genetic variables was performed using Pearson’s chi-square test or applying Fisher’s exact test for the qualitative variables. For the quantitative variables, Student’s *t*-test was applied to the variables that complied with normality, and the Mann-Whitney U test was applied for non-normal variables. The statistical power of genetic association was determined using G*Power 3.1.9.7. [35].

Multivariate analysis (logistic or linear regression) was used to calculate the adjusted odds ratio (OR) and a 95% confidence interval (CI_95%_) for the potential EULAR response, LDA and remission prognostic factors. The goodness of fit for each model was analyzed with the Hosmer–Lemeshow test and the omnibus test of coefficients, as well as calculating the Cox–Snell and Nagelkerke r^2^ coefficients. All tests were two-sided, with a probability of 0.05 or less being considered statistically significant, and were performed using R 3.5.1 or PLINK toolset free-access software for whole genome association analysis [36,37,38].

The Hardy–Weinberg equilibrium and the haplotype frequencies were determined and Lewontin’s D-prime (D’) and the linkage disequilibrium coefficient (r^2^) were calculated.

The linkage disequilibrium (LD) for each polymorphism was calculated with the PLINK genome association analysis program [39]. The analysis of the haplotype frequencies and their association with the responses analyzed in the study was performed using the snpStats program, a web-based tool for the analysis of association studies [40,41,42,43,44].

## 3. Results

### 3.1. Patient Characteristics

The clinical and sociodemographic characteristics of the 109 patients included in the study are described in Table 1. Of all the patients, 71.56% were women (78/109); the mean age at RA diagnosis was 44.94 ± 14.46 years and the median disease duration was 16 [8,9,10,11,12,13,14,15,16,17,18,19,20,21,22] years; 13.76% (15/109) were smokers. All the patients had been treated with other DMARDs, for a median period of 36 [12,13,14,15,16,17,18,19,20,21,22,23,24,25,26,27,28,29,30,31,32,33,34,35,36,37,38,39,40,41,42,43,44,45,46,47,48,49,50,51,52,53,54,55,56,57,58,59,60] months, and the median number of previous biological treatments was 2 [1,2,3]. A total of 14.68% (16/109) were ABA-bionaive, 26.61% (29/109) began the treatment after failure of 1 tumor necrosis factor inhibitor (TNFi), 28.44% (31/109) did so after failure of 2 TNFis and 30.28% (33/109) began treatment with ABA after failure of 3 or more TNFis. The mean age at the start of ABA treatment was 56.37 ± 13.05 years, and the median duration of ABA therapy was 28 [14,15,16,17,18,19,20,21,22,23,24,25,26,27,28,29,30,31,32,33,34,35,36,37,38,39,40,41,42,43,44,45,46] months. ABA was administered by SC route in 55.05% (60/109) and by IV route in 44.95% (49/109). A total of 49.54% (54/109) patients received concomitant csDMARDs: MTX (34.86%; 38/109) and LFN (12.84%; 14/109). Glucocorticoids were administered in 83.49% of cases (91/109). The reason for suspension of ABA treatment was primary failure in 22.94% of cases (25/109), secondary failure in 8.26% (9/109) and adverse reaction in 5.50% of patients (6/109).

Positive RF and ACPAs values occurred in 79.82% (87/109) and 72.48% (79/109) of cases, respectively. The description of the clinical parameters, such as DAS28 and HAQ, and the acute phase reactants (CRP and ESR) are shown in Table 1.

### 3.2. Clinical Effectiveness of ABA

The effectiveness of ABA after 6 and 12 months of treatment was evaluated in 105 (96.33%) and 92 (84.40%) patients, respectively, out of the total study population of 109 patients (Table 2). The remaining patients (3.67%; 4/109) discontinued the treatment before 6 months, two because of loss of effectiveness (1.83%; 2/109) and another two because of adverse reactions (1.83%; 2/109). A further 12.38% (13/105) discontinued the treatment before 12 months, 10.48% (11/105) due to loss of effectiveness and 1.90% (2/105) due to adverse reactions (Table 1).

The EULAR response was satisfactory at 6 months in 34.29% (36/105) of cases and increased to 46.74% (43/92) after 12 months of treatment. Disease remission status increased from 17.14% (18/105) at 6 months to 30.43% (28/92) at 12 months. There was LDA in 20.95% (22/105) of patients at 6 months and in 21.74% (20/92) at 12 months.

In ABA-bionaive patients, the EULAR response was satisfactory in 53.33% (8/15) of cases after 6 months of treatment, and this increased to 78.57% (11/14) after 12 months of treatment. Of these patients, 64.29% (9/14) attained remission after 12 months with ABA. The findings for LDA were 33.33% (5/15) at 6 months and 21.43% (3/14) at 12 months. All these results are shown in detail in Table 2.

### 3.3. Genotype Distribution

**Figure 1:**
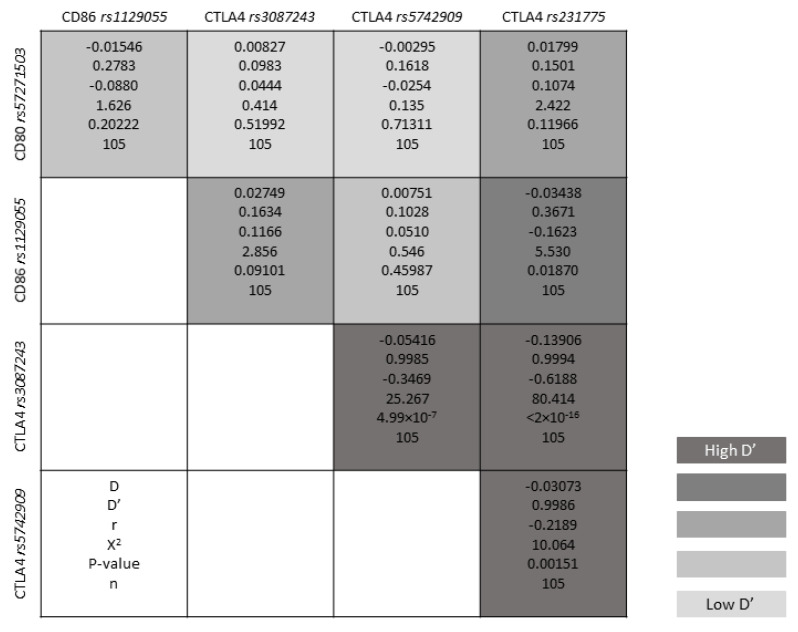
Linkage disequilibrium.

Genotype frequencies matched expected values as per the Hardy–Weinberg equilibrium (HWE) model (Table S1). The D’ linkage disequilibrium (LD) and r^2^ values are given in Table S2, and Figure 1 shows a graph for LD. Polymorphisms *CTLA-4 rs3087243* and *CTLA-4 rs231775* are in linkage disequilibrium (r^2^ = 0.42635; D’ = 1; Table S2). All the polymorphisms showed minor allele frequencies higher than 1% and none of them has been excluded from the analysis (Table S3). Haplotype frequencies estimation values are given in Tables S4–S9. Haplotype analysis adjusted by duration of previous BTs, PVAS and DAS28 revealed that the haplotypes GAACA (OR = 46.73; CI_95%_ = 3.32–658.10), GGGTA (OR = 54.83; CI_95%_ = 1.55–1933.70), GGGCA (OR = 368.25; CI_95%_ = 6.52–20,796.54) and AGGCG (OR = 56.35; CI_95%_ = 1.29–2464.20) at 6 months of ABA therapy; and the haplotype analysis adjusted by PVAS shows that the haplotype GGGCA (OR = 34.85; CI_95%_ = 2.61–465.09) at 12 months for *CD80 (rs57271503), CD86 (rs1129055), CTLA-4 (rs3087243), CTLA-4 (rs5742909)* and *CTLA-4 (rs231775)* were associated with a higher EULAR response (Table S10; Table S13). Moreover, haplotype analysis adjusted by baseline DAS28 revealed that the haplotypes GAACA (OR = 9.56; CI_95%_ = 1.62–56.28), GGGCA (OR = 70.76; CI_95%_ = 2.44–2049.74), GGGTA (OR = 35.97; CI_95%_ = 1.15–1125.40), GAGTA (OR = Inf; CI_95%_ = Inf–Inf), GAGCA (OR = 0.04; CI_95%_ = 0.00–0.91) and AAACA (OR = Inf; CI_95%_ = Inf–Inf) at 6 months of ABA administration; and the haplotype GGGCG (OR = 0.20; CI_95%_ = 0.04–0.93) at 12 months for *CD80 (rs57271503), CD86 (rs1129055), CTLA-4 (rs3087243), CTLA-4 (rs5742909)* and *CTLA-4 (rs231775)* were associated with LDA (Tables S11 and S14). Haplotype associations with response are presented in Tables S10–S15.

### 3.4. ABA Response Predictors at 6 Months

#### 3.4.1. EULAR Response

In the bivariate analysis, a higher EULAR response was found in patients with fewer years of disease duration, who were administered the drug as monotherapy, without concomitant GCs and who had been receiving previous bDMARDs treatment for a shorter period. As for clinical variables, patients responded better to the therapy when their initial basal values for DAS28, NPJ, NIJ, PVAS, ESR and HAQ were lower (values are detailed in Table S16). The multivariate analysis showed that the independent variables associated with EULAR response at 6 months were lower duration of previous bDMARDs (OR = 0.97; CI_95%_ = 0.95–0.99) and lower baseline values of the DAS28 (OR = 0.52; CI_95%_ = 0.30–0.87) and PVAS (OR = 0.95; CI_95%_ = 0.91–0.98) clinical variables. The multivariate analysis results are shown in Table 3.

#### 3.4.2. Low Disease Activity (LDA)

In the bivariate analysis, LDA was found to be associated with a shorter period of treatment with previous bDMARDs and with administration of ABA by the SC route. Low disease activity was also associated with lower baseline levels of DAS28, PVAS and HAQ (values are detailed in Table S17). The *CTLA-4 rs5742909-T* allele showed a tendency toward association with LDA (*T* vs. *CC*; OR = 6.67; CI_95%_ = 0.84–52.74; Table S17). After performing the multivariate analysis, we found that a lower initial DAS28 value (OR = 0.69; CI_95%_ = 0.49–0.96; Table 3) indicated a tendency to a better therapeutic response.

#### 3.4.3. Remission

In the bivariate analysis, greater disease remission was found in patients who were not administered GCs or other concomitant csDMARDs, who had been receiving ABA therapy for longer and whose baseline levels of DAS28, NPJ, NIJ, PVAS, ESR and HAQ were lower. The *CTLA-4 rs3087243-G* allele (*G *vs.* AA*; *p* = 0.006) and *CTLA-4 rs231775-G* (*G *vs.* AA*; OR = 3.06; CI_95%_ = 1.05–10.20) were found to be associated with remission of the disease (values are detailed in Table S18). In the multivariate analysis, a significant association was found between duration of treatment with ABA (OR = 1.05; CI_95%_ = 1.01–1.08; Table 3), non-administration of concomitant GCs (OR = 7.34; CI_95%_ = 1.33–54.79; Table 3) and lower baseline values for the NPJ (OR = 0.65; CI_95%_ = 0.48–0.81) and ESR (OR = 0.89; CI_95%_ = 0.82–0.95; Table 3) clinical variables.

### 3.5. ABA Response Predictors at 12 Months

#### 3.5.1. EULAR Response

In the bivariate analysis, a significant association was found between satisfactory EULAR response and non-administration of concomitant GCs bionaive patients and lower baseline levels of DAS28, NPJ, NIJ, PVAS and HAQ (values are detailed in Table S16). An association was found between the *CTLA-4 rs3087243-G* allele (*G *vs.* AA*; OR = 4.41; CI_95%_ = 1.56–14.59; Table S16) and *CTLA-4 rs231775-G* allele (*G *vs.* AA*, OR = 2.71; CI_95%_ = 1.17–6.44; Table S16) and a satisfactory EULAR response at 12 months. The *CTLA-4 rs5742909* (*CT*>*CC*>*TT*; *p* = 0.038) showed a tendency toward association with a satisfactory EULAR response (*p* = 0.066). In the multivariate analysis, the EULAR response was greater in patients with lower baseline PVAS (OR = 0.93; CI_95%_ = 0.90–0.96), the *CTLA-4 rs5742909-T* allele (*T *vs.* CC*; OR = 5.88; CI_95%_ = 1.48–23.29) and the *CTLA-4 rs231775-G* allele (*G *vs.* AA*; OR = 3.48; CI_95%_ = 1.20–10.09) (values are detailed in Table 3).

#### 3.5.2. Low Disease Activity (LDA)

In the bivariate and multivariate analysis, an association was found between LDA at 12 months and the *CTLA-4 rs5742909-T* allele (*T *vs.* CC*; OR = 4.75; CI_95%_ = 1.35–17.94; Table 3), and the *CTLA-4 rs231775-G* allele (*G *vs.* AA*, OR = 4.64; CI_95%_ = 1.49–17.94; Table 3). A tendency was found toward a significant association between LDA and the *CTLA-4 rs3087243-G* allele (*G* vs. *AA*; OR = 3.66; CI_95%_ = 0.76–35.37; Table S17).

#### 3.5.3. Remission

In the bivariate analysis, disease remission was related to shorter disease duration, bionaive patients and also shorter duration of previous BTs, as well as a lower baseline value of the NPJ, NIJ, PVAS and HAQ clinical variables (values are detailed in Table S18). No association was found between the polymorphisms studied and remission of the disease at 12 months. The multivariate analysis showed a significant association with earlier age at the start of ABA therapy (OR = 0.96; CI_95%_ = 0.92–0.99; Table 3), lower number of previous BTs (OR = 0.56; CI_95%_ = 0.34–0.92; Table 3) and lower baseline PVAS (OR = 0.95; CI_95%_ = 0.93–0.98; Table 3).

## 4. Discussion

Abatacept is a biologic drug used as a first-line treatment in patients diagnosed with RA who have not reached a state of disease remission after administration of previous DMARDs. Interindividual variability in response to ABA is very wide in patients with RA. Our study included 105 patients diagnosed with RA and treated with ABA as first-/second-/third-or-more-line therapy for at least 6 months, and 92 maintained the treatment for at least 12 months. As regards the effectiveness of ABA, bionaive patients showed a higher EULAR response and greater disease remission after ABA treatment compared to patients who had already undergone previous BTs (78.57% vs. 46.74%; 64.29% vs. 30.43%; after 12 months of ABA). Our results are consistent with a study performed in 2716 Caucasian RA patients treated with ABA, in which the bionaive patients showed a higher EULAR response after 6 (OR-adjusted = 3.59, CI_95%_ = 2.25–5.72) and 12 months (OR-adjusted = 4.29, CI_95%_ = 2.77–6.65) of ABA treatment [45]. Moreover, in the multivariate analysis of our results, the number and duration of previous BTs were significantly associated with remission of the disease and EULAR response after 12 and 6 months of treatment, respectively. Numerous observational studies and clinical trials have reached similar conclusions [46,47,48,49]. This hypothesis may be justified, given that the low effectiveness of BTs in many patients causes autoimmune and inflammatory processes to be triggered more rapidly, leading to a gradual and irreversible progression of the disease. Thus, BTs used in advanced phases of the disease lose their efficacy due to the extensive development of the pathology. Hence the importance of diagnosing the disease early and looking for biomarkers to help select the most effective therapy for each patient. Regarding biochemical markers, several studies have described the presence of positive ACPA as constituting an ABA treatment response factor by inhibiting T-lymphocyte activation, and consequently autoantibody production [50,51]. A study based on the “Orencia and Rheumatoid Arthritis” registry, which included 1003 Caucasian patients with RA, showed significant differences in ACPA values between patients with satisfactory and unsatisfactory EULAR responses (75.9% vs. 62.2%: *p* = 0.001) [51]. However, our study showed a tendency to a significant relationship between positive ACPA values and EULAR response after 6 months of treatment with ABA.

Polymorphisms in the genes associated with the drug’s mechanism of action could explain the substantial variability of response in patients treated with ABA. In this study, we determined the role of SNPs on the *CD80*, *CD86* and *CTLA-4* genes in the treatment response. ABA is a fusion protein that acts by selectively modulating T-lymphocyte activation [52]. This modulation is performed physiologically by CTLA-4 [52]. Several SNPs located on this gene have been investigated for their association with autoimmune diseases [22,23,28,53]. The *CTLA-4 rs5742909* polymorphism (*C*>*T*) is in the promoter region of exon 1 of the *CTLA-4* gene, affecting the ATG transcription initiation codon [54]. This SNP could alter the transcription factor binding site [22,55,56,57]. In addition, the *CTLA-4 rs5742909-T* allele has been linked to increased RNAm expression and higher levels of the CTLA-4 protein [53,58]. This theory supports the results obtained in our study, since the *CTLA-4 rs5742909-T* allele was associated with a higher EULAR response and lower LDA ratios after 12 months of ABA treatment, in the multivariate analysis. This SNP is presumably related to protection against autoimmunity and progression of the inflammatory response, which would explain the fact that patients who carry it show a greater response to ABA treatment [56,59]. A previous study analyzed the relationship of ABA with the *CTLA-4 rs5742909* polymorphism in 39 Caucasian patients after 6 months of treatment, finding no significant association (*p* > 0.05) [18]. A study conducted in 200 Caucasian patients diagnosed with RA found higher levels of CTLA-4 in those carrying the *CTLA-4 rs5742909-CT* genotype (*CT* vs. *CC*; *p* < 0.001) [60]. Another of the *CTLA-4* gene polymorphisms that have been studied is *rs3087243* (*G*>*A*), located in exon 4 in the 3′UTR region [61]. Previous research has associated this SNP with a change in the levels of RNAm expression of the CTLA-4 molecule, also affecting its various isoforms [62,63]. It has also been observed that it could affect the peptide glycosylation level, resulting in a non-functional CTLA-4 molecule [23]. Specifically, the *CTLA-4 rs3087243-G* allele has been associated with a reduction in CTLA-4 production [62,64]. In our study, patients carrying the *CTLA-4 rs3087243-G* allele showed a higher EULAR response and greater LDA at 12 months. Furthermore, the *CTLA-4 rs3087243-GG* (*GG* vs. *AA*/*AG*) genotype was associated with greater remission of the disease after 6 months of treatment with ABA in the bivariate analysis; there was no significant association in the multivariate analysis. A study carried out in 200 patients with RA and 200 healthy Caucasian controls showed an association between the *CTLA-4 rs3087243-GG* and *CTLA-4 rs3087243-GA* genotypes and higher CTLA-4 levels in healthy controls (*p* < 0.001) [60]. Moreover, no previous study has shown significant results relating to the association between the *CTLA-4 rs3087243* SNP and response to ABA [18]. Another of the SNPs studied was *CTLA-4 rs231775* (*A* > *G*). In this case, a change from Thr to Ala occurs at position +49 of exon 1 of the gene [65]. This change of amino acid could cause incomplete glycosylation of the protein in the endoplasmic reticulum, which would affect production of the soluble form of the molecule. Previous research has shown a reduction in CTLA-4 production associated with the *CTLA-4 rs231775-GG* genotype [66,67,68]. In our study, we found that patients carrying the *CTLA-4 rs231775-G* allele showed a higher EULAR response as well as greater LDA after 12 months of treatment with ABA in the multivariate analysis. Remission of the disease after 6 months of treatment was also greater in patients with the *CTLA-4 rs231775-G* allele in the bivariate analysis, but in this case the association was not maintained in the multivariate analysis. In a study conducted in 715 Caucasian patients with multiple sclerosis and 527 healthy controls, it was observed that lesser CTLA-4 expression was associated with the least aggressive form of the disease, which could be explained by the fact that the CTLA-4 molecule promotes an increase in reservoirs of memory T lymphocytes, because it reduces induced T-lymphocyte apoptosis [69,70]. Consequently, lesser CTLA-4 production would result in a reduction in memory T lymphocytes, leading to less inflammation and a lower probability of inflammatory relapses [69]. Given the large inflammatory component involved in the development of RA, this hypothesis could explain the greater therapeutic response in patients who express lower levels of CTLA-4.

The polymorphisms associated with the *CTLA-4* gene have also been investigated for their association with the risk of developing autoimmune diseases [22,23,28,53]. In various previous studies, the *CTLA-4 rs5742909-T* allele has been associated with a lower risk of suffering from autoimmune diseases such as RA, systemic lupus erythematosus or Graves’ disease [53,56,58,59,71,72]. The *CTLA-4 rs3087243* polymorphism has also been associated with a risk of suffering from autoimmune pathologies. A study on scleritis carried out in 446 Asian patients and 710 healthy controls associated both the *CTLA-4 rs3087243-GG* genotype (OR = 1.55; CI_95%_ = 1.19–2.01) and the *CTLA-4 rs3087243-G* allele (OR = 1.46; CI_95%_ = 1.18–1.85) with a higher risk of developing the disease, compared with healthy controls [28]. As for the *CTLA-4 rs231775* SNP, Muñoz–Valle et al. found that the *CTLA-4 rs231775-G* allele was a risk factor for suffering from RA (46.8% vs. 37.7%, OR = 1.45; *p* = 0.01) [73]. Likewise, Downie et al. showed an association between the *CTLA-4 rs231775-A* allele and susceptibility to Sjögren’s syndrome in 111 Caucasian patients and 156 controls (*p* = 0.032) [64].

Abatacept binds selectively to the CD80/CD86 complex, like CTLA-4, preventing the latter from binding to the CD28 receptor and thereby suppressing T-cell activation [52]. Polymorphisms in these proteins could produce conformational changes in the receptor, affecting its interaction with ABA and causing the therapeutic response. The *CD86 rs1129055* polymorphism (*G* > *A*) could give rise to a change in the protein’s phosphorylation level, influencing the process of negative regulation of T-lymphocyte proliferation that occurs after its interaction with CTLA-4 [21,74]. In our study, we did not find a significant association with the response variables studied. Our results are consistent with the only previous study carried out in 39 Caucasian patients, which analyzed the association between the *CD86 rs1129055* and therapeutic response to ABA, since in this case, too, no significant relationship with EULAR response was found after 6 months of ABA treatment [18]. Similarly, no association was found between the *CD80 rs57271503* polymorphism and therapeutic response to ABA in our patients after 6 and 12 months of treatment. These results are in line with those obtained in the previous study comprising 39 Caucasian patients diagnosed with RA, which analyzed the relationship between the *CD80 rs57271503* SNP and EULAR response [18]. No other studies have evaluated the role of these SNPs in the effectiveness of ABA in patients diagnosed with RA.

The limitations of our study include the limited sample size, which could be responsible for the loss of statistically significant association for the *CD80 rs57271503* and *CD86 rs1129055* SNPs. Thus, our sample size has a statistical power to detect genetic association of 60%. Nevertheless, all the patients were recruited from the same hospital cohort, following the same therapeutic protocols, by the same team of rheumatologists, which ensured the homogeneity and reliability of the clinical variables collected. All patients diagnosed during the study period were recruited, ensuring the representativeness of the sample. Despite the limited sample size, the effects observed in these patients were clear. Further studies in larger cohorts will be needed to confirm the prognostic value of the biomarkers, particularly the polymorphisms of the *CTLA-4*, *CD80* and *CD86* genes.

These results suggest that the *CTLA-4 rs3087243*, *rs231775* and *rs5742909* polymorphisms could act as predictors of response to ABA treatment in patients diagnosed with RA.

## 5. Conclusions

Patients with RA treated with ABA showed a better EULAR response and LDA rates when they had the *CTLA-4 rs5742909-T* or *CTLA-4 rs231775-G* polymorphisms. As regards the clinical variables, greater remission was observed after 12 months of treatment in patients who initiated ABA treatment earlier, with a lower number of previous BTs and a lower PVAS value at the start of treatment. Likewise, shorter duration of previous BTs is associated with a higher EULAR response after 6 months of treatment with ABA.

## Figures and Tables

**Table 1:** Demographic and clinical characteristics of patients treated with abatacept.

Variable	Baseline		
	N	%	Mean ± Standard Deviation
**Sex**			
Female	78	71.56	-
Male	31	28.44	-
**Smoking**			
Smokers	15	13.76	-
Former-smokers	11	10.09	-
Non smokers	83	76.15	-
**Age at RA diagnosis**	109	-	44.94 ± 14.46
**Disease duration (years)**	109	-	16 (8–22)
**Age at ABA start**	109	-	56.37 ± 13.05
**Duration of ABA (months)**	109	-	28 (14–46)
**ABA administration**			
Subcutaneous	60	55.05	-
Intravenous	49	44.95	-
**Concomitant csDMARDs**			
Methotrexate	38	34.86	-
Leflunomide	14	12.84	-
Others	2	1.83	-
None	55	50.45	
**Concomitant glucocorticoids**			
No	18	16.51	-
Yes	91	83.49	-
**Monotherapy**			
No	103	94.50	-
Yes	6	5.50	-
**Number of previous BTs**	109	-	2 (1–3)
**Duration of previous BTs (months)**	109	-	36 (12–60)
**Previous BTs**			
Bionaive	16	14.68	-
1 TNFi	29	26.61	-
2 TNFis	31	28.44	-
3 or more TNFis	33	30.28	-
**Suspension reason of ABA**			
Primary failure	25	22.94	-
Secondary failure	9	8.26	-
Adverse reaction	6	5.50	-
No suspension	69	63.30	-
**Rheumatoid factor**			
Negative	22	20.18	-
Positive	87	79.82	-
**ACPAs**			
Negative	30	27.52	-
Positive	79	72.48	-
**DAS28**	109	-	4.77 ± 1.43
**NPJ**	109	-	7 (3–10)
**NIJ**	109	-	3 (1–6)
**PVAS**	109	-	70 (50–80)
**CRP**	109	-	2.42 (1.40–5.00)
**ESR**	109	-	22 (10–38)
**HAQ**	109	-	1.75 (1.25–2.00)

ABA, abatacept; ACPAs, anti-cyclic citrullinated peptide antibodies; BT, biological therapy; CRP, C-reactive protein; csDMARDs, conventional synthetic disease-modifying antirheumatic drugs; DAS28, 28-joints Disease Activity Score; ESR, erythrocyte sedimentation rate; HAQ, Health Assessment Questionnaire score; NIJ, number of inflamed joints; NPJ, number of painful joints; PVAS, patient’s visual analogue scale; RA, rheumatoid arthritis; TNFi, tumor necrosis factor inhibitor. Qualitative variables are shown as number (percentage, %). Quantitative variables with a normal distribution are shown as mean ± standard deviation. Quantitative variables with a non-normal distribution are shown as p_50_ (p_25_–p_75_).

**Table 2:** Clinical Effectiveness of Abatacept in non-bionaive and ABA-bionaive patients.

Non-Bionaive Patients				
Response variable	6 months		12 months	
	N	%	N	%
**EULAR response**				
Satisfactory	36	34.29	43	46.74
Unsatisfactory	69	65.71	49	53.26
**Remission (DAS28 < 2.6)**	18	17.14	28	30.43
**LDA (2.6 ≥ DAS28 ≥ 3.2)**	22	20.95	20	21.74
ABA-Bionaive Patients				
Response variable		6 months		12 months
	N	%	N	%
**EULAR response**				
Satisfactory	8	53.33	11	78.57
Unsatisfactory	7	46.67	3	21.43
**Remission (DAS28 < 2.6)**	3	20	9	64.29
**LDA (2.6 ≥ DAS28 ≥ 3.2)**	5	33.33	3	21.43

ABA, abatacept; EULAR, European League Against Rheumatism; LDA, Low-activity disease.

**Table 3:** Predictors of response at 6 and 12 months of treatment with abatacept in rheumatoid arthritis patients (multivariate analysis).

Response Variable	Independent Variable	B	Odds Ratio	*p*-Value (Variable)	95% Confidence INTERVAL	R^2^	Goodness of Fit
**6 months**							
**EULAR response**							
	Duration of previous BTs (months)	−0.026	0.97	0.004	0.95–0.99	R^2^ Cox Snell = 0.382 R^2^ Nagelkerke = 0.528	X^2^ = 10.396 *p* = 0.238
	PVAS	−0.052	0.95	0.003	0.91–0.98		
	DAS28	−0.651	0.52	0.015	0.30–0.87		
**LDA**							
	DAS28	−0.367	0.69	0.032	0.49–0.96	R^2^ Cox Snell = 0.045 R^2^ Nagelkerke = 0.070	X^2^ = 7.062 *p* = 0.529
**Remission**							
	Duration of ABA (months)	0.047	1.05	0.002	1.01–1.08	R^2^ Cox Snell = 0.349 R^2^ Nagelkerke = 0.581	X^2^ = 62.774 *p* < 0.001
	Concomitant glucocorticoids	1.993	7.34	0.031	1.33–54.79		
	NPJ	−0.436	0.65	0.001	0.48–0.81		
	ESR	−0.109	0.89	0.004	0.82–0.95		
**12 months**							
**EULAR response**							
	PVAS	−0.069	0.93	<0.001	0.90–0.96	R^2^ Cox Snell = 0.335 R^2^ Nagelkerke = 0.447	X^2^ = 2.509 *p* = 0.961
	*CTLA-4 rs5742909 (T* vs. *CC)*	1.772	5.88	0.012	1.48–23.29		
	*CTLA-4 rs231775 (G* vs. *AA)*	1.247	3.48	0.022	1.20–10.09		
**LDA**							
	*CTLA-4 rs5742909 (T* vs. *CC)*	1.556	4.75	0.016	1.35–17.94	R^2^ Cox Snell = 0.117 R^2^ Nagelkerke = 0.180	X^2^ = 0.156 *p* = 1
	*CTLA-4 rs231775 (G* vs. *AA)*	1.540	4.67	0.013	1.49–17.94		
**Remission**							
	Age at ABA start	−0.044	0.96	0.027	0.92–0.99	R^2^ Cox Snell = 0.239 R^2^ Nagelkerke = 0.339	X^2^ = 6.561 *p* = 0.585
	Number of previous BTs	−0.574	0.56	0.023	0.34–0.92		
	PVAS	−0.051	0.95	<0.001	0.93–0.98		

ABA, abatacept; BT, biological therapy; DAS28, 28-joints Disease Activity Score; ESR, erythrocyte sedimentation rate; EULAR, European League Against Rheumatism; LDA, low disease activity; NPJ, number of painful joints; PVAS, patient’s visual analogue scale.

## Supplementary Materials

The following are available online at https://www.mdpi.com/2075-4426/10/4/220/s1, Figure S1: Linkage disequilibrium, Table S1: Hardy-Weinberg equilibrium, Table S2: Linkage disequilibrium, Table S3: Minor allele frequencies of SNPs, Table S4: Haplotype frequencies estimation EULAR response at 6 months ABA, Table S5: Haplotype frequencies estimation LDA at 6 months ABA, Table S6: Haplotype frequencies estimation remission at 6 months ABA, Table S7: Haplotype frequencies estimation EULAR response at 12 months ABA, Table S8: Haplotype frequencies estimation LDA at 12 months ABA, Table S9: Haplotype frequencies estimation remission at 12 months ABA, Table S10: Haplotype association with EULAR response at 6 months ABA adjusted by ACPA, duration of previous BTs, PVAS and DAS28, Table S11: Haplotype association with LDA at 6 months ABA adjusted by baseline DAS28, Table S12: Haplotype association with remission at 6 months ABA adjusted by ABA duration, concomitant glucocorticoids, NPJ and ESR, Table S13: Haplotype association with EULAR response at 12 months ABA adjusted by PVAS, Table S14: Haplotype association with LDA at 12 months ABA, Table S15: Haplotype association with remission at 12 months ABA adjusted by age at ABA start, number of previous BTs, PVAS, Table S16: Predictors of EULAR response at 6 and 12 months of treatment with abatacept in rheumatoid arthritis patients (bivariate analysis), Table S17: Predictors of LDA at 6 and 12 months of treatment with abatacept in rheumatoid arthritis patients (bivariate analysis), Table S18: Predictors of remission at 6 and 12 months of treatment with abatacept in rheumatoid arthritis patients (bivariate analysis).

## Abbreviations

ABA	Abatacept
ACPA	anti-cyclic citrullinated peptide antibodies
ACR	American College of Rheumatology
bDMARDs	Biologic disease-modifying antirheumatic drugs
BT	biological therapy
CRP	C-reactive protein
csDMARDs	conventional synthetic disease-modifying antirheumatic drugs
CTLA-4	cytotoxic T-lymphocyte-associated antigen 4
DAS28	28-joints Disease Activity Score
DMARDs	disease-modifying antirheumatic drugs
ESR	erythrocyte sedimentation rate
EULAR	European League Against Rheumatism
GC	glucocorticoid
HAQ	Health Assessment Questionnaire score
IV	intravenous
LDA	Low-activity disease
LFN	leflunomide
MTX	methotrexate
NIJ	number of inflamed joints
NPJ	number of painful joints
PVAS	patient’s visual analogue scale
RA	rheumatoid arthritis
RF	rheumatoid factor
SC	subcutaneous
TNFi	tumor necrosis factor inhibitor
tsDMARDs	targeted synthetic disease-modifying antirheumatic drugs
