# Supplementary material for: Impact of Single-Nucleotide Polymorphisms of CTLA-4, CD80 and CD86 on the Effectiveness of Abatacept in Patients with Rheumatoid Arthritis
Source: J Pers Med. 2020 Nov 11;10(4):220. doi: 10.3390/jpm10040220 (PMC7711575; doi:10.3390/jpm10040220)
Supplement: Supplementary file 1 [file jpm-10-00220-s001.zip › Table S1.docx]

**Table S1. Hardy-Weinberg Equilibrium**

| **Chr** | **SNP** | **Sample** | **Minor**  **Allele** | **Major**  **Allele** | **Genotype counts** | **Observed heterozygosity** | **Expected heterozygosity** | **p-value** |
| --- | --- | --- | --- | --- | --- | --- | --- | --- |
| 2 | *rs3087243* | Cases | G | A | 29/51/29 | 0.4679 | 0.500 | 0.565 |
| 2 | *rs5742909* | Cases | T | C | 2/19/88 | 0.1743 | 0.189 | 0.329 |
| 2 | *rs231775* | Cases | G | A | 6/49/54 | 0.4495 | 0.403 | 0.340 |
| 3 | *rs57271503* | Cases | A | G | 2/31/76 | 0.2844 | 0.269 | 0.734 |
| 3 | *rs1129055* | Cases | A | G | 12/49/48 | 0.4495 | 0.446 | 1 |
| *Chr, Chromosome* | | | | | | | | |
