# Supplementary material for: Impact of Single-Nucleotide Polymorphisms of CTLA-4, CD80 and CD86 on the Effectiveness of Abatacept in Patients with Rheumatoid Arthritis
Source: J Pers Med. 2020 Nov 11;10(4):220. doi: 10.3390/jpm10040220 (PMC7711575; doi:10.3390/jpm10040220)
Supplement: Supplementary file 1 [file jpm-10-00220-s001.zip › Table S2.docx]

**Table S2. Linkage disequilibrium**

| **Chr** | **BP** | **SNP** | **Chr** | **BP** | **SNP** | **R^2^** | **D** |
| --- | --- | --- | --- | --- | --- | --- | --- |
| 2 | 203874196 | *rs3087243* | 2 | 203867991 | *rs231775* | 0.42635 | 1 |
| *Chr, Chromosome; BP, Physical position (base-pair)* | | | | | | | |
