# Supplementary material for: Impact of Single-Nucleotide Polymorphisms of CTLA-4, CD80 and CD86 on the Effectiveness of Abatacept in Patients with Rheumatoid Arthritis
Source: J Pers Med. 2020 Nov 11;10(4):220. doi: 10.3390/jpm10040220 (PMC7711575; doi:10.3390/jpm10040220)
Supplement: Supplementary file 1 [file jpm-10-00220-s001.zip › Table S3.docx]

Table S3. Minor allele frequencies of SNPs.

| **Chr** | **SNP** | **Minor Allele** | **Major Allele** | **MAF** |
| --- | --- | --- | --- | --- |
| 2 | *rs3087243* | G | A | 0.500 |
| 2 | *rs5742909* | T | C | 0.106 |
| 2 | *rs231775* | G | A | 0.279 |
| 3 | *rs57271503* | A | G | 0.161 |
| 3 | *rs1129055* | A | G | 0.335 |
| *Chr, Chromosome; MAF, Minor allele frequency* | | | | |
