# Supplementary material for: Impact of Single-Nucleotide Polymorphisms of CTLA-4, CD80 and CD86 on the Effectiveness of Abatacept in Patients with Rheumatoid Arthritis
Source: J Pers Med. 2020 Nov 11;10(4):220. doi: 10.3390/jpm10040220 (PMC7711575; doi:10.3390/jpm10040220)
Supplement: Supplementary file 1 [file jpm-10-00220-s001.zip › Table S5.docx]

**Table S5. Haplotype frequencies estimation LDA at 6 months ABA**

|  | ***CD80***  ***rs57271503*** | ***CD86***  ***rs1129055*** | ***CTLA4***  ***rs3087243*** | ***CTLA4***  ***rs5742909*** | ***CTLA4***  ***rs231775*** | **Total** | **No LDA** | **LDA** | **Cumulative frequency** |
| --- | --- | --- | --- | --- | --- | --- | --- | --- | --- |
| 1 | G | G | A | C | A | 0.237 | 0.205 | 0.353 | 0.237 |
| 2 | G | A | A | C | A | 0.169 | 0.189 | 0.107 | 0.406 |
| 3 | G | G | G | C | G | 0.162 | 0.177 | 0.100 | 0.568 |
| 4 | G | G | G | C | A | 0.073 | 0.082 | 0.024 | 0.642 |
| 5 | A | G | A | C | A | 0.069 | 0.062 | 0.108 | 0.711 |
| 6 | G | G | G | T | A | 0.066 | 0.083 | 0.023 | 0.777 |
| 7 | A | G | G | C | G | 0.059 | 0.054 | 0.074 | 0.836 |
| 8 | G | A | G | C | G | 0.058 | 0.046 | 0.099 | 0.894 |
| 9 | G | A | G | T | A | 0.036 | 0.039 | NA | 0.929 |
| 10 | G | A | G | C | A | 0.033 | 0.017 | 0.112 | 0.962 |
| 11 | A | A | A | C | A | 0.019 | 0.019 | NA | 0.982 |
| * | A | A | G | C | A | 0.008 | 0.009 | 0 | 0.989 |
| * | A | A | G | T | A | 0.008 | 0.011 | NA | 0.998 |
| * | A | A | G | C | G | 0.002 | 0.007 | NA | 1 |
| * | A | G | G | T | A | 0 | 0 | NA | 1 |
| **Rare haplotypes* | | | | | | | | | |
