# Supplementary material for: Impact of Single-Nucleotide Polymorphisms of CTLA-4, CD80 and CD86 on the Effectiveness of Abatacept in Patients with Rheumatoid Arthritis
Source: J Pers Med. 2020 Nov 11;10(4):220. doi: 10.3390/jpm10040220 (PMC7711575; doi:10.3390/jpm10040220)
Supplement: Supplementary file 1 [file jpm-10-00220-s001.zip › Table S6.docx]

**Table S6. Haplotype frequencies estimation remission at 6 months ABA**

|  | ***CD80***  ***rs57271503*** | ***CD86***  ***rs1129055*** | ***CTLA4***  ***rs3087243*** | ***CTLA4***  ***rs5742909*** | ***CTLA4***  ***rs231775*** | **Total** | **No**  **remission** | **Remission** | **Cumulative frequency** |
| --- | --- | --- | --- | --- | --- | --- | --- | --- | --- |
| 1 | G | G | A | C | A | 0.2367 | 0.2723 | 0.1067 | 0.2367 |
| 2 | G | A | A | C | A | 0.1692 | 0.1894 | NA | 0.406 |
| 3 | G | G | G | C | G | 0.1622 | 0.1218 | 0.3306 | 0.5681 |
| 4 | G | G | G | C | A | 0.0734 | 0.0711 | 0.0332 | 0.6415 |
| 5 | A | G | A | C | A | 0.0698 | 0.0569 | 0.035 | 0.7114 |
| 6 | G | G | G | T | A | 0.0655 | 0.0641 | 0.1032 | 0.7769 |
| 7 | A | G | G | C | G | 0.059 | 0.0806 | NA | 0.8359 |
| 8 | G | A | G | C | G | 0.0578 | 0.0563 | NA | 0.8937 |
| 9 | G | A | G | T | A | 0.0359 | 0.0261 | 0.0306 | 0.9296 |
| 10 | G | A | G | C | A | 0.0326 | 0.0381 | 0.1389 | 0.9622 |
| 11 | A | A | A | C | A | 0.0195 | 0.016 | 0.0727 | 0.9816 |
| * | A | A | G | C | A | 0.0083 | 0 | 0 | 0.9899 |
| * | A | A | G | T | A | 0.0081 | 0.0075 | NA | 0.998 |
| * | A | A | G | C | G | 0.0019 | NA | NA | 1 |
| * | A | G | G | T | A | 0 | 0 | NA | 1 |
| **Rare haplotypes* | | | | | | | | | |
