# Supplementary material for: Impact of Single-Nucleotide Polymorphisms of CTLA-4, CD80 and CD86 on the Effectiveness of Abatacept in Patients with Rheumatoid Arthritis
Source: J Pers Med. 2020 Nov 11;10(4):220. doi: 10.3390/jpm10040220 (PMC7711575; doi:10.3390/jpm10040220)
Supplement: Supplementary file 1 [file jpm-10-00220-s001.zip › Table S8.docx]

**Table S8. Haplotype frequencies estimation LDA at 12 months ABA**

|  | ***CD80***  ***rs57271503*** | ***CD86***  ***rs1129055*** | ***CTLA4***  ***rs3087243*** | ***CTLA4***  ***rs5742909*** | ***CTLA4***  ***rs231775*** | **Total** | **No LDA** | **LDA** | **Cumulative frequency** |
| --- | --- | --- | --- | --- | --- | --- | --- | --- | --- |
| 1 | G | G | A | C | A | 0.232 | 0.228 | 0.167 | 0.232 |
| 2 | G | A | A | C | A | 0.178 | 0.181 | 0.258 | 0.411 |
| 3 | G | G | G | C | G | 0.156 | 0.160 | 0.189 | 0.567 |
| 4 | G | G | G | C | A | 0.074 | 0.094 | NA | 0.641 |
| 5 | A | G | G | C | G | 0.068 | 0.033 | 0.144 | 0.708 |
| 6 | A | G | A | C | A | 0.064 | 0.097 | NA | 0.772 |
| 7 | G | A | G | C | G | 0.061 | 0.053 | 0.036 | 0.834 |
| 8 | G | G | G | T | A | 0.058 | 0.031 | 0.175 | 0.892 |
| 9 | G | A | G | T | A | 0.036 | 0.042 | 0 | 0.927 |
| 10 | G | A | G | C | A | 0.031 | 0.037 | NA | 0.958 |
| 11 | A | A | A | C | A | 0.019 | 0 | NA | 0.978 |
| * | A | A | G | C | A | 0.009 | 0.022 | NA | 0.988 |
| * | A | A | G | T | A | 0.009 | NA | 0 | 0.997 |
| * | A | A | G | C | G | 0.003 | 0.011 | 0.031 | 1 |
| * | A | G | G | C | A | 0 | NA | NA | 1 |
| * | A | G | G | T | A | 0 | 0.003 | NA | 1 |
| **Rare haplotypes* | | | | | | | | | |
