# Supplementary material for: Impact of Single-Nucleotide Polymorphisms of CTLA-4, CD80 and CD86 on the Effectiveness of Abatacept in Patients with Rheumatoid Arthritis
Source: J Pers Med. 2020 Nov 11;10(4):220. doi: 10.3390/jpm10040220 (PMC7711575; doi:10.3390/jpm10040220)
Supplement: Supplementary file 1 [file jpm-10-00220-s001.zip › Table S9.docx]

**Table S9. Haplotype frequencies estimation remission at 12 months ABA**

|  | ***CD80***  ***rs57271503*** | ***CD86***  ***rs1129055*** | ***CTLA4***  ***rs3087243*** | ***CTLA4***  ***rs5742909*** | ***CTLA4***  ***rs231775*** | **Total** | **No remission** | **Remission** | **Cumulative frequency** |
| --- | --- | --- | --- | --- | --- | --- | --- | --- | --- |
| 1 | G | G | A | C | A | 0.2323 | 0.2016 | 0.2143 | 0.2323 |
| 2 | G | A | A | C | A | 0.1783 | 0.2263 | 0.1071 | 0.4106 |
| 3 | G | G | G | C | G | 0.156 | 0.144 | 0.2152 | 0.5667 |
| 4 | G | G | G | C | A | 0.0739 | 0.0708 | 0.1071 | 0.6405 |
| 5 | A | G | G | C | G | 0.0678 | 0.0882 | 0 | 0.7083 |
| 6 | A | G | A | C | A | 0.064 | 0.0721 | 0.0866 | 0.7723 |
| 7 | G | A | G | C | G | 0.0614 | 0.0412 | 0.1062 | 0.8337 |
| 8 | G | G | G | T | A | 0.0581 | 0.056 | 0.0732 | 0.8919 |
| 9 | G | A | G | T | A | 0.0356 | 0.0377 | 0.0339 | 0.9274 |
| 10 | G | A | G | C | A | 0.0305 | 0.0349 | 0 | 0.9579 |
| 11 | A | A | A | C | A | 0.0199 | 0 | 0.0563 | 0.9778 |
| * | A | A | G | C | A | 0.0098 | 0.0193 | NA | 0.9876 |
| * | A | A | G | T | A | 0.0096 | 0 | NA | 0.9972 |
| * | A | A | G | C | G | 0.0028 | 0 | NA | 1 |
| * | A | G | G | C | A | 0 | NA | 0 | 1 |
| * | A | G | G | T | A | 0 | 0 | NA | 1 |
| **Rare haplotypes* | | | | | | | | | |
