# Supplementary material for: Impact of Single-Nucleotide Polymorphisms of CTLA-4, CD80 and CD86 on the Effectiveness of Abatacept in Patients with Rheumatoid Arthritis
Source: J Pers Med. 2020 Nov 11;10(4):220. doi: 10.3390/jpm10040220 (PMC7711575; doi:10.3390/jpm10040220)
Supplement: Supplementary file 1 [file jpm-10-00220-s001.zip › Table S10.docx]

**Table S10. Haplotype association with EULAR response at 6 months ABA adjusted by duration of previous BTs, PVAS and DAS28**

|  | ***CD80***  ***rs57271503*** | ***CD86***  ***rs1129055*** | ***CTLA4***  ***rs3087243*** | ***CTLA4***  ***rs5742909*** | ***CTLA4***  ***rs231775*** | **Frequencies** | **Odds ratio (CI_95%_)** | **p-value** |
| --- | --- | --- | --- | --- | --- | --- | --- | --- |
| 1 | G | G | A | C | A | 0.236 | 1.00 | - |
| 2 | G | A | A | C | A | 0.174 | 46.73 (3.32 - 658.10) | 0.005 |
| 3 | G | G | G | C | G | 0.149 | 1.03 (0.16 - 6.43) | 0.980 |
| 4 | G | G | G | T | A | 0.076 | 54.83 (1.55 - 1933.70) | 0.030 |
| 5 | G | G | G | C | A | 0.076 | 368.25 (6.52 - 20796.54) | 0.005 |
| 6 | G | A | G | C | G | 0.069 | 0.95 (0.04 - 23.66) | 0.970 |
| 7 | A | G | A | C | A | 0.067 | 0.42 (0.01 - 12.12) | 0.620 |
| 8 | A | G | G | C | G | 0.064 | 56.35 (1.29 - 2464.20) | 0.039 |
| 9 | G | A | G | C | A | 0.031 | 0.12 (0.01 - 2.97) | 0.200 |
| 10 | G | A | G | T | A | 0.023 | 0.01 (0.00 - 1.54) | 0.075 |
| 11 | A | A | A | C | A | 0.019 | 3.62 (0.01 - 2007.23) | 0.690 |
| * | - | - | - | - | - | 0.018 | 0.95 (0.00-224.02) | 0.990 |
|  | **Rare haplotype*. CI_95%_, 95% Confidence interval. *Global haplotype association p-value: 0.0022* | | | | | | | |
