# Supplementary material for: Impact of Single-Nucleotide Polymorphisms of CTLA-4, CD80 and CD86 on the Effectiveness of Abatacept in Patients with Rheumatoid Arthritis
Source: J Pers Med. 2020 Nov 11;10(4):220. doi: 10.3390/jpm10040220 (PMC7711575; doi:10.3390/jpm10040220)
Supplement: Supplementary file 1 [file jpm-10-00220-s001.zip › Table S11.docx]

**Table S11. Haplotype association with LDA at 6 months ABA adjusted by baseline DAS28**

|  | ***CD80***  ***rs57271503*** | ***CD86***  ***rs1129055*** | ***CTLA4***  ***rs3087243*** | ***CTLA4***  ***rs5742909*** | ***CTLA4***  ***rs231775*** | **Frequencies** | **Odds ratio (CI_95%_)** | **p-value** |
| --- | --- | --- | --- | --- | --- | --- | --- | --- |
| 1 | G | G | A | C | A | 0.2349 | 1.00 | - |
| 2 | G | A | A | C | A | 0.1714 | 9.56 (1.62 - 56.28) | 0.014 |
| 3 | G | G | G | C | G | 0.1576 | 3.70 (0.47 - 29.09) | 0.220 |
| 4 | G | G | G | C | A | 0.076 | 70.76 (2.44 - 2049.74) | 0.015 |
| 5 | A | G | A | C | A | 0.0722 | 1.26 (0.10 - 16.06) | 0.860 |
| 6 | G | G | G | T | A | 0.0701 | 35.97 (1.15 - 1125.40) | 0.044 |
| 7 | G | A | G | C | G | 0.0632 | 0.75 (0.12 - 4.58) | 0.750 |
| 8 | A | G | G | C | G | 0.0558 | 2.14 (0.17 - 27.23) | 0.560 |
| 9 | G | A | G | T | A | 0.0312 | Inf (Inf-Inf) | <0.001 |
| 10 | G | A | G | C | A | 0.0288 | 0.04 (0.00 - 0.91) | 0.047 |
| 11 | A | A | A | C | A | 0.0167 | Inf (Inf-Inf) | <0.001 |
| * | - | - | - | - | - | 0.022 | 22.70 (0.00-167041.02) | 0.490 |
|  | **Rare haplotype.* CI_95%_, 95% Confidence interval; Inf, infinite. *Global haplotype association p-value: 0.012* | | | | | | | |
