# Supplementary material for: Impact of Single-Nucleotide Polymorphisms of CTLA-4, CD80 and CD86 on the Effectiveness of Abatacept in Patients with Rheumatoid Arthritis
Source: J Pers Med. 2020 Nov 11;10(4):220. doi: 10.3390/jpm10040220 (PMC7711575; doi:10.3390/jpm10040220)
Supplement: Supplementary file 1 [file jpm-10-00220-s001.zip › Table S12.docx]

**Table S12. Haplotype association with remission at 6 months ABA adjusted by duration of ABA, concomitant glucocorticoids, NPJ and ESR**

|  | ***CD80***  ***rs57271503*** | ***CD86***  ***rs1129055*** | ***CTLA4***  ***rs3087243*** | ***CTLA4***  ***rs5742909*** | ***CTLA4***  ***rs231775*** | **Frequencies** | **Odds ratio (CI_95%_)** | **p-value** |
| --- | --- | --- | --- | --- | --- | --- | --- | --- |
| 1 | G | G | A | C | A | 0.247 | 1.00 | - |
| 2 | G | A | A | C | A | 0.166 | 0.00 (Inf-Inf) | 1 |
| 3 | G | G | G | C | G | 0.149 | - | - |
|  | CI_95%_, 95% Confidence interval; Inf, infinite. | | | | | | | |
