# Supplementary material for: Impact of Single-Nucleotide Polymorphisms of CTLA-4, CD80 and CD86 on the Effectiveness of Abatacept in Patients with Rheumatoid Arthritis
Source: J Pers Med. 2020 Nov 11;10(4):220. doi: 10.3390/jpm10040220 (PMC7711575; doi:10.3390/jpm10040220)
Supplement: Supplementary file 1 [file jpm-10-00220-s001.zip › Table S13.docx]

**Table S13. Haplotype association with EULAR response at 12 months ABA adjusted by PVAS**

|  | ***CD80***  ***rs57271503*** | ***CD86***  ***rs1129055*** | ***CTLA4***  ***rs3087243*** | ***CTLA4***  ***rs5742909*** | ***CTLA4***  ***rs231775*** | **Frequencies** | **Odds ratio (CI_95%_)** | **p-value** |
| --- | --- | --- | --- | --- | --- | --- | --- | --- |
| 1 | G | G | A | C | A | 0.251 | 1.00 | - |
| 2 | G | A | A | C | A | 0.186 | 1.97 (0.44 - 8.78) | 0.380 |
| 3 | G | G | G | C | G | 0.146 | 0.42 (0.09 - 2.10) | 0.300 |
| 4 | A | G | G | C | G | 0.073 | 1.54 (0.16 - 14.44) | 0.710 |
| 5 | G | G | G | C | A | 0.066 | 34.85 (2.61 - 465.09) | 0.009 |
| 6 | G | G | G | T | A | 0.061 | 0.33 (0.04 - 3.00) | 0.330 |
| 7 | G | A | G | C | G | 0.055 | 0.70 (0.10 - 4.85) | 0.720 |
| 8 | A | G | A | C | A | 0.039 | 6.83 (0.11 - 436.83) | 0.370 |
| 9 | G | A | G | C | A | 0.033 | 9.19 (0.07 - 1164.48) | 0.370 |
| 10 | G | A | G | T | A | 0.027 | 2.99 (0.10 - 89.31) | 0.530 |
| 11 | A | A | A | C | A | 0.018 | - | - |
|  | CI_95%_, 95% Confidence interval; Inf, infinite. | | | | | | | |
