# Supplementary material for: Impact of Single-Nucleotide Polymorphisms of CTLA-4, CD80 and CD86 on the Effectiveness of Abatacept in Patients with Rheumatoid Arthritis
Source: J Pers Med. 2020 Nov 11;10(4):220. doi: 10.3390/jpm10040220 (PMC7711575; doi:10.3390/jpm10040220)
Supplement: Supplementary file 1 [file jpm-10-00220-s001.zip › Table S14.docx]

**Table S14. Haplotype association with LDA at 12 months ABA**

|  | ***CD80***  ***rs57271503*** | ***CD86***  ***rs1129055*** | ***CTLA4***  ***rs3087243*** | ***CTLA4***  ***rs5742909*** | ***CTLA4***  ***rs231775*** | **Frequencies** | **Odds ratio (CI_95%_)** | **p-value** |
| --- | --- | --- | --- | --- | --- | --- | --- | --- |
| 1 | G | G | A | C | A | 0.224 | 1.00 | - |
| 2 | G | A | A | C | A | 0.187 | 0.24 (0.05 - 1.10) | 0.070 |
| 3 | G | G | G | C | G | 0.163 | 0.20 (0.04 - 0.93) | 0.043 |
| 4 | G | G | G | C | A | 0.073 | - | - |
|  | CI_95%_, 95% Confidence interval | | | | | | | |
