# Supplementary material for: Impact of Single-Nucleotide Polymorphisms of CTLA-4, CD80 and CD86 on the Effectiveness of Abatacept in Patients with Rheumatoid Arthritis
Source: J Pers Med. 2020 Nov 11;10(4):220. doi: 10.3390/jpm10040220 (PMC7711575; doi:10.3390/jpm10040220)
Supplement: Supplementary file 1 [file jpm-10-00220-s001.zip › Table S15.docx]

**Table S15. Haplotype association with remission at 12 months ABA adjusted by age at ABA start, number of previous BTs, PVAS**

|  | ***CD80***  ***rs57271503*** | ***CD86***  ***rs1129055*** | ***CTLA4***  ***rs3087243*** | ***CTLA4***  ***rs5742909*** | ***CTLA4***  ***rs231775*** | **Frequencies** | **Odds ratio (CI_95%_)** | **p-value** |
| --- | --- | --- | --- | --- | --- | --- | --- | --- |
| 1 | G | G | A | C | A | 0.218 | 1.00 | - |
| 2 | G | A | A | C | A | 0.186 | 4.28 (0.72 - 25.54) | 0.120 |
| 3 | G | G | G | C | G | 0.164 | 1.18 (0.22 - 6.20) | 0.850 |
| 4 | G | G | G | C | A | 0.074 | 4.72 (0.64 - 34.80) | 0.130 |
| 5 | A | G | A | C | A | 0.071 | 1.93 (0.12 - 32.06) | 0.650 |
| 6 | A | G | G | C | G | 0.064 | 21.10 (0.04 - 10964.75) | 0.340 |
| 7 | G | G | G | T | A | 0.062 | 1.87 (0.14 - 25.01) | 0.640 |
| 8 | G | A | G | C | G | 0.059 | 0.83 (0.11 - 6.35) | 0.850 |
| 9 | G | A | G | T | A | 0.032 | 0.71 (0.03 - 15.27) | 0.830 |
| 10 | G | A | G | C | A | 0.031 | 4.67 (0.16 - 139.58) | 0.380 |
| 11 | A | A | A | C | A | 0.020 | 0.61 (0.01 - 39.53) | 0.820 |
| * | - | - | - | - | - | 0.019 | 3.70 (0.03-395.82) | 0.580 |
|  | **Rare haplotypes*. CI_95%_, 95% Confidence interval. *Global haplotype association p-value: 0.66* | | | | | | | |
