# Supplementary material for: Impact of Single-Nucleotide Polymorphisms of CTLA-4, CD80 and CD86 on the Effectiveness of Abatacept in Patients with Rheumatoid Arthritis
Source: J Pers Med. 2020 Nov 11;10(4):220. doi: 10.3390/jpm10040220 (PMC7711575; doi:10.3390/jpm10040220)
Supplement: Supplementary file 1 [file jpm-10-00220-s001.zip › Table S16.docx]

**Table S16. Predictors of EULAR response at 6 and 12 months of treatment with abatacept in rheumatoid arthritis patients (bivariate analysis)**

|  | **6 months** | | | | | | | **12 months** | | | | | |  |
| --- | --- | --- | --- | --- | --- | --- | --- | --- | --- | --- | --- | --- | --- | --- |
| **Independent variable** | **EULAR response** | | | | | | **Independent variable** | **EULAR response** | | | | | |  |
|  | **N** | **Unsatisfactory** | **Satisfactory** | **p-value** | **OR** | **CI_95%_** |  | **N** | **Unsatisfactory** | **Satisfactory** | **p-value** | **OR** | **CI_95%_** |  |
| **Sex** |  |  |  |  |  |  | **Sex** |  |  |  |  |  |  |  |
| **Female** | 76 | 51 (67.1) | 25 (32.9) | 0.627 | - | **-** | **Female** | 64 | 37 (57.8) | 27 (42.9) | 0.186 | - | - |  |
| **Male** | 29 | 11 (62.1) | 11 (37.9) |  |  |  | **Male** | 28 | 12 (42.2) | 16 (57.1) |  |  |  |  |
| **Smoking** |  |  |  |  |  |  | **Smoking** |  |  |  |  |  |  |  |
| **Smokers** | 15 | 9 (60) | 6 (40) | 0.885 | - | **-** | **Smokers** | 13 | 6 (46.2) | 7 (53.8) | 0.936 | - | - |  |
| **Former-smokers** | 11 | 7 (63.6) | 4 (36.4) |  |  |  | **Former-smokers** | 9 | 5 (55.6) | 4 (44.4) |  |  |  |  |
| **Non-smokers** | 79 | 53 (67.1) | 26 (32.9) |  |  |  | **Non-smokers** | 70 | 38 (54.3) | 32 (45.7) |  |  |  |  |
| **Age at RA diagnosis** | 105 | 45.33 ± 13.75 | 44.75 ± 15.34 | 0.843 | - | **-** | **Age at RA diagnosis** | 92 | 44.65 ± 14.17 | 45.84 ± 15.27 | 0.701 |  |  |  |
| **Disease duration (years)** | 105 | 17 (10 - 23) | 11 (7 - 18) | 0.029 | 0.94 | 0.89 - 0.99 | **Disease duration (years)** | 92 | 17 (10 - 24) | 12 (8 - 19) | 0.077 | 0.95 | 0.91 - 1.00 |  |
| **Age at ABA start** | 105 | 57.65 ± 13.08 | 53.97 ± 13.22 | 0.176 | - | **-** | **Age at ABA start** | 92 | 57.24 ± 13.71 | 55.60 ± 13.69 | 0.568 | - | - |  |
| **Duration of ABA (months)** | 105 | 24 (13 - 45) | 32.50 (16.75 - 50.50) | 0.174 | - | **-** | **Duration of ABA (months)** | 92 | 29 (18 - 53) | 33 (23 - 60) | 0.305 | - | - |  |
| **ABA administration** |  |  |  |  |  |  | **ABA administration** |  |  |  |  |  |  |  |
| **Subcutaneous** | 57 | 34 (59.6) | 23 (40.4) | 0.154 | - | **-** | **Subcutaneous** | 50 | 24 (48) | 26 (52) | 0.269 | - | - |  |
| **Intravenous** | 48 | 35 (72.9) | 13 (27.1) |  |  |  | **Intravenous** | 42 | 25 (59.5) | 17 (40.5) |  |  |  |  |
| **Concomitant csDMARDs** |  |  |  |  |  |  | **Concomitant csDMARDs** |  |  |  |  |  |  |  |
| **Methotrexate** | 36 | 25 (69.4) | 11 (30.6) | 0.488 | - | **-** | **Methotrexate** | 32 | 18 (56) | 14 (43.8) | 0.734 | - | - |  |
| **Leflunomide** | 14 | 8 (57.1) | 6 (42.9) |  |  |  | **Leflunomide** | 13 | 7 (53.8) | 6 (46.2) |  |  |  |  |
| **Others** | 2 | 1 (50) | 1 (50) |  |  |  | **Others** | 2 | 1 (50) | 1 (50) |  |  |  |  |
| **Concomitant glucocorticoids** |  |  |  |  |  |  | **Concomitant glucocorticoids** |  |  |  |  |  |  |  |
| **Yes** | 89 | 63 (70.8) | 26 (29.2) | 0.009 | 3.98 | 1.17 - 14.80 | **Yes** | 78 | 45 (57.7) | 33 (42.3) | 0.044 | 3.41 | 0.98 - 11.82 |  |
| **No** | 16 | 6 (37.5) | 10 (62.5) |  |  |  | **No** | 14 | 4 (28.6) | 10 (71.4) |  |  |  |  |
| **Monotherapy** |  |  |  |  |  |  | **Monotherapy** |  |  |  |  |  |  |  |
| **No** | 99 | 68 (68.7) | 31 (31.3) | 0.017 | 10.71 | 1.13 - 524.60 | **No** | 86 | 48 (55.8) | 38 (44.2) | 0.063 | 6.32 | 0.71 - 56.37 |  |
| **Yes** | 6 | 1 (16.7) | 5 (83.3) |  |  |  | **Yes** | 6 | 1 (16.7) | 5 (83.3) |  |  |  |  |
| **Number of previous BTs** | 105 | 2 (1 - 3) | 1.5 (1 - 3) | 0.254 | - | - | **Number of previous BTs** | 92 | 2 (1 - 2) | 2 (0.5 - 3) | 0.406 | - | - |  |
| **Duration of previous BTs (months)** | 105 | 48 (24 - 60) | 24 (7.5 - 36) | 0.006 | 0.98 | 0.96 - 0.99 | **Duration of previous BTs (months)** | 92 | 24 (12 - 60) | 24 (3 - 48) | 0.149 | - | - |  |
| **Previous BTs** |  |  |  |  |  |  | **Previous BTs** |  |  |  |  |  |  |  |
| **Bionaive** | 15 | 7 (46.7) | 8 (53.3) | 0.227 | - | **-** | **Bionaive** | 14 | 3 (21.4) | 11 (78.6) | 0.013 | 5.50 | 1.33 - 29.15 |  |
| **1 TNFi** | 28 | 18 (64.3) | 10 (35.7) |  |  |  | **1 TNFi** | 25 | 15 (60) | 10 (40) |  |  |  |  |
| **2 TNFis** | 31 | 24 (77.4) | 7 (22.6) |  |  |  | **2 TNFis** | 28 | 20 (71.4) | 8 (28.6) |  |  |  |  |
| **3 or more TNFis** | 31 | 20 (64.5) | 11 (35.5) |  |  |  | **3 or more TNFis** | 25 | 11 (44) | 14 (56) |  |  |  |  |
| **Rheumatoid factor** |  |  |  |  |  |  | **Rheumatoid factor** |  |  |  |  |  |  |  |
| **Negative** | 22 | 14 (63.6) | 8 (36.4) | 0.817 | - | **-** | **Negative** | 20 | 10 (50) | 10 (50) | 0.741 | - | - |  |
| **Positive** | 83 | 55 (66.3) | 28 (33.7) |  |  |  | **Positive** | 72 | 39 (54.2) | 33 (45.8) |  |  |  |  |
| **ACPAs** |  |  |  |  |  |  | **ACPAs** |  |  |  |  |  |  |  |
| **Negative** | 29 | 23 (79.3) | 6 (20.7) | 0.069 | 2.48 | 0.85 - 8.34 | **Negative** | 24 | 13 (54.2) | 11 (45.8) | 0.917 | - | - |  |
| **Positive** | 76 | 46 (60.5) | 30 (39.5) |  |  |  | **Positive** | 68 | 36 (52.9) | 32 (47.1) |  |  |  |  |
| **DAS28** | 105 | 5.29 ± 1.13 | 3.78 ± 1.50 | <0.001 | 0.39 | 0.25 - 0.58 | **DAS28** | 92 | 5.13 ± 1.00 | 4.24 ± 1.64 | 0.002 | 0.60 | 0.42 - 0.83 |  |
| **NPJ** | 105 | 8 (5 - 11) | 3 (0.75 - 6.25) | <0.001 | 0.82 | 0.72 - 0.91 | **NPJ** | 92 | 8 (5 - 11) | 5 (2 - 8) | 0.005 | 0.92 | 0.83 - 0.99 |  |
| **NIJ** | 105 | 4 (2 - 6) | 1 (0 - 3) | <0.001 | 0.77 | 0.64 - 0.89 | **NIJ** | 92 | 3 (2 - 6) | 1 (0 - 5) | 0.025 | 0.89 | 0.76 - 1.01 |  |
| **PVAS** | 105 | 70 (70 - 80) | 50 (27.5 - 60) | <0.001 | 0.93 | 0.90 - 0.95 | **PVAS** | 92 | 70 (70 - 80) | 50 (30 - 70) | <0.001 | 0.94 | 0.91 - 0.96 |  |
| **CRP** | 105 | 2.36 (1.40 - 4.30) | 2.85 (1.26 - 5.13) | 0.805 | - | **-** | **CRP** | 92 | 2.36 (1.4 - 5.5) | 2.39 (1.5 - 4.2) | 0.848 | - | - |  |
| **ESR** | 105 | 27 (14 - 45) | 14.5 (8 - 31.25) | 0.019 | 0.97 | 0.95 - 0.99 | **ESR** | 92 | 22 (12 - 54) | 22 (9.5 - 35) | 0.338 |  |  |  |
| **HAQ** | 105 | 2 (1.60 - 2.13) | 1.2 (0.75 - 1.85) | <0.001 | 0.25 | 0.11 - 0.49 | **HAQ** | 92 | 1.88 ± 0.63 | 1.32 ± 0.73 | <0.001 | 0.29 | 0.14 - 0.56 |  |
| ***CD80 rs57271503*** |  |  |  |  |  |  | ***CD80 rs57271503*** |  |  |  |  |  |  |  |
| ***AA*** | 2 | 1 (50.0) | 1 (50.0) | 0.561 | - | **-** | ***AA*** | 2 | 2 (100) | 0 (0) | 0.310 | - | - |  |
| ***GG*** | 72 | 49 (68.1) | 23 (31.9) |  |  |  | ***GG*** | 62 | 34 (54.8) | 28 (45.2) |  |  |  |  |
| ***AG*** | 31 | 19 (61.3) | 12 (38.7) |  |  |  | ***AG*** | 28 | 13 (46.4) | 15 (53.6) |  |  |  |  |
| ***A*** | 33 | 20 (60.6) | 13 (39.4) | 0.455 | - | **-** | ***A*** | 30 | 15 (50) | 15 (50) | 0.663 | - | - |  |
| ***G*** | 103 | 68 (66) | 35 (34) | 1 | - | **-** | ***G*** | 90 | 47 (52.2) | 43 (47.8) | 0.180 | - | - |  |
| ***CD86 rs1129055*** |  |  |  |  |  |  | ***CD86 rs1129055*** |  |  |  |  |  |  |  |
| ***AA*** | 11 | 6 (54.5) | 5 (45.5) | 0.336 | - | **-** | ***AA*** | 11 | 6 (54.5) | 5 (45.5) | 0.481 | - | - |  |
| ***GG*** | 46 | 28 (60.9) | 18 (39.1) |  |  |  | ***GG*** | 39 | 18 (46.2) | 21 (53.8) |  |  |  |  |
| ***AG*** | 48 | 35 (72.9) | 13 (27.1) |  |  |  | ***AG*** | 42 | 25 (59.5) | 17 (40.5) |  |  |  |  |
| ***A*** | 59 | 41 (69.5) | 18 (30.5) | 0.356 | - | **-** | ***A*** | 53 | 31 (58.5) | 22 (41.5) | 0.241 | - | - |  |
| ***G*** | 94 | 63 (67.0) | 31 (33.0) | 0.505 | - | **-** | ***G*** | 81 | 43 (53.1) | 38 (46.9) | 0.927 | - | - |  |
| ***CTLA4 rs3087243*** |  |  |  |  |  |  | ***CTLA4 rs3087243*** |  |  |  |  |  |  |  |
| ***AA*** | 27 | 21 (77.8) | 6 (22.2) | 0.210 | - | **-** | ***AA*** | 23 | 18 (78.3) | 5 (21.7) | 0.006 | - | - |  |
| ***GG*** | 28 | 19 (67.9) | 9 (32.1) |  |  |  | ***GG*** | 24 | 14 (58.3) | 10 (41.7) |  |  |  |  |
| ***AG*** | 50 | 29 (58.0) | 21 (42.0) |  |  |  | ***AG*** | 45 | 17 (37.8) | 28 (62.2) |  |  |  |  |
| ***A*** | 77 | 50 (64.9) | 27 (35.1) | 0.780 | - | **-** | ***A*** | 68 | 35 (51.5) | 33 (48.5) | 0.562 | - | - |  |
| ***G*** | 78 | 48 (61.5) | 30 (38.5) | 0.126 | - | **-** | ***G*** | 69 | 31 (44.9) | 38 (55.1) | 0.005 | 4.41 | 1.56 - 14.59 |  |
| ***CTLA4 rs5742909*** |  |  |  |  |  |  | ***CTLA4 rs5742909*** |  |  |  |  |  |  |  |
| ***CC*** | 84 | 54 (64.3) | 30 (35.7) | 0.641 | - | **-** | ***CC*** | 75 | 43 (57.3) | 32 (42.7) | 0.038 | - | - |  |
| ***TT*** | 2 | 1 (50.0) | 1 (50.0) |  |  |  | ***TT*** | 2 | 2 (100) | 0 (0) |  |  |  |  |
| ***CT*** | 19 | 14 (73.7) | 5 (26.3) |  |  |  | ***CT*** | 15 | 4 (26.7) | 11 (73.3) |  |  |  |  |
| ***C*** | 103 | 68 (66.0) | 35 (34.0) | 0.636 | - | **-** | ***C*** | 90 | 47 (52.2) | 43 (47.8) | 0.180 | - | - |  |
| ***T*** | 21 | 15 (71.4) | 6 (28.6) | 0.537 | - | **-** | ***T*** | 17 | 6 (35.3) | 11 (64.7) | 0.100 | 2.46 | 0.84 - 7.81 |  |
| ***CTLA4 rs231775*** |  |  |  |  |  |  | ***CTLA4 rs231775*** |  |  |  |  |  |  |  |
| ***AA*** | 52 | 37 (71.2) | 15 (28.8) | 0.468 | - | **-** | ***AA*** | 44 | 29 (65.9) | 15 (34.1) | 0.066 | - | - |  |
| ***GG*** | 6 | 4 (66.7) | 2 (33.3) |  |  |  | ***GG*** | 5 | 2 (40) | 3 (60) |  |  |  |  |
| ***AG*** | 47 | 28 (59.6) | 19 (40.4) |  |  |  | ***AG*** | 43 | 18 (41.9) | 25 (58.1) |  |  |  |  |
| ***A*** | 99 | 65 (65.7) | 34 (34.3) | 0.959 | - | **-** | ***A*** | 87 | 47 (54) | 40 (46) | 0.662 | - | - |  |
| ***G*** | 53 | 32 (60.4) | 21 (39.6) | 0.245 | - | **-** | ***G*** | 48 | 20 (41.7) | 28 (58.3) | 0.023 | 2.71 | 1.17 - 6.44 |  |
| ABA, abatacept; ACPAs, anti - cyclic citrullinated peptide antibodies; BT, biological therapy; CI_95%_, 95% Confidence interval; CRP, C-reactive protein; csDMARDs, conventional synthetic disease-modifying antirheumatic drugs; DAS28, 28 - joints Disease Activity Score; ESR, erythrocyte sedimentation rate; EULAR, European League Against Rheumatism; HAQ, Health Assessment Questionnaire score; NIJ, number of inflamed joints; NPJ, number of painful joints; OR, Odds ratio; PVAS, patient’s visual analogue scale; RA, rheumatoid arthritis; TNFi, tumor necrosis factor inhibitor. | | | | | | | | | | | | | |  |
