# Supplementary material for: Impact of Single-Nucleotide Polymorphisms of CTLA-4, CD80 and CD86 on the Effectiveness of Abatacept in Patients with Rheumatoid Arthritis
Source: J Pers Med. 2020 Nov 11;10(4):220. doi: 10.3390/jpm10040220 (PMC7711575; doi:10.3390/jpm10040220)
Supplement: Supplementary file 1 [file jpm-10-00220-s001.zip › Table S17.docx]

**Table S17. Predictors of LDA at 6 and 12 months of treatment with abatacept in rheumatoid arthritis patients (bivariate analysis)**

|  | **6 months** | | | | | | | **12 months** | | | | | |  |
| --- | --- | --- | --- | --- | --- | --- | --- | --- | --- | --- | --- | --- | --- | --- |
| **Independent variable** | **LDA** | | | | | | **Independent variable** | **LDA** | | | | | |  |
|  | **N** | **No LDA** | **LDA** | **p-value** | **OR** | **CI_95%_** |  | **N** | **No LDA** | **LDA** | **p-value** | **OR** | **CI_95%_** |  |
| **Sex** |  |  |  |  |  |  | **Sex** |  |  |  |  |  |  |  |
| **Female** | 76 | 60 (78.9) | 16 (21.1) | 0.967 | - | - | **Female** | 64 | 51 (79.7) | 13 (20.3) | 0.616 | - | - |  |
| **Male** | 29 | 23 (79.3) | 6 (20.7) |  |  |  | **Male** | 28 | 21 (75) | 7 (25) |  |  |  |  |
| **Smoking** |  |  |  |  |  |  | **Smoking** |  |  |  |  |  |  |  |
| **Smokers** | 15 | 13 (86.7) | 2 (13.3) | 0.720 | - | - | **Smokers** | 13 | 10 (76.9) | 3 (23.1) | 0.169 | - | - |  |
| **Former-smokers** | 11 | 8 (72.7) | 3 (27.3) |  |  |  | **Former-smokers** | 9 | 5 (55.6) | 4 (44.4) |  |  |  |  |
| **Non-smokers** | 79 | 62 (78.5) | 17 (21.5) |  |  |  | **Non-smokers** | 70 | 57 (81.4) | 13 (18.6) |  |  |  |  |
| **Age at RA diagnosis** | 105 | 45.12 ± 14.38 | 45.18 ± 14.08 | 0.986 | - | - | **Age at RA diagnosis** | 92 | 44.04 ± 14.58 | 49.40 ± 14.36 | 0.148 | - | - |  |
| **Disease duration (years)** | 105 | 16 (9-22) | 13 (6.25-20.25) | 0.177 | - | - | **Disease duration (years)** | 92 | 14 (8-22) | 16.5 (10.75-20.50) | 0.513 | - | - |  |
| **Age at ABA start** | 105 | 56.87 ± 13.34 | 54.59 ± 12.70 | 0.474 | - | - | **Age at ABA start** | 92 | 55.07 ± 13.93 | 61.55 ± 11.54 | 0.059 | 1.04 | 0.99-1.08 |  |
| **Duration of ABA (months)** | 105 | 28 (15-54) | 29 (15.5-42.5) | 0.981 | - | - | **Duration of ABA (months)** | 92 | 29 (19-50) | 34.5 (27.5-70) | 0.199 | - | - |  |
| **ABA administration** |  |  |  |  |  |  | **ABA administration** |  |  |  |  |  |  |  |
| **Subcutaneous** | 57 | 41 (71.9) | 16 (28.1) | 0.051 | 2.71 | 0.89-9.31 | **Subcutaneous** | 50 | 40 (80) | 10 (20) | 0.659 | - | - |  |
| **Intravenous** | 48 | 42 (87.5) | 6 (12.5) |  |  |  | **Intravenous** | 42 | 32 (76.2) | 10 (23.8) |  |  |  |  |
| **Concomitant csDMARDs** |  |  |  |  |  |  | **Concomitant csDMARDs** |  |  |  |  |  |  |  |
| **Methotrexate** | 36 | 31 (86.1) | 5 (13.9) | 0.304 | - | - | **Methotrexate** | 32 | 25 (78.1) | 7 (21.9) | 0.953 | - | - |  |
| **Leflunomide** | 14 | 11 (78.6) | 3 (21.4) |  |  |  | **Leflunomide** | 13 | 11 (84.6) | 2 (15.4) |  |  |  |  |
| **Others** | 2 | 1 (50) | 1 (50) |  |  |  | **Others** | 1 | 1 (100) | 0 (0) |  |  |  |  |
| **Concomitant glucocorticoids** |  |  |  |  |  |  | **Concomitant glucocorticoids** |  |  |  |  |  |  |  |
| **Yes** | 89 | 71 (79.8) | 18 (20.2) | 0.739 | - | - | **Yes** | 78 | 63 (80.3) | 15 (19.2) | 0.176 | - | - |  |
| **No** | 16 | 12 (75) | 4 (25) |  |  |  | **No** | 14 | 9 (64.3) | 5 (35.7) |  |  |  |  |
| **Monotherapy** |  |  |  |  |  |  | **Monotherapy** |  |  |  |  |  |  |  |
| **No** | 99 | 78 (78.8) | 21 (21.2) | 0.791 | - | - | **No** | 86 | 69 (80.2) | 17 (19.8) | 0.114 | - | - |  |
| **Yes** | 6 | 5 (83.3) | 1 (16.7) |  |  |  | **Yes** | 6 | 3 (50) | 3 (50) |  |  |  |  |
| **Number of previous BTs** | 105 | 2 (1-3) | 2 (1-3) | 0.730 | - | - | **Number of previous BTs** | 92 | 2 (1-3) | 2 (1-3) | 0.732 | - | - |  |
| **Duration of previous BTs (months)** | 105 | 36 (24-60) | 24 (9-33) | 0.048 | 0.98 | 0.96-0.99 | **Duration of previous BTs (months)** | 92 | 24 (12-60) | 36 (24-51.25) | 0.199 | - | - |  |
| **Previous BTs** |  |  |  |  |  |  | **Previous BTs** |  |  |  |  |  |  |  |
| **Bionaive** | 15 | 10 (66.7) | 5 (33.3) | 0.352 | - | - | **Bionaive** | 14 | 11 (78.6) | 3 (21.4) | 0.989 | - | - |  |
| **1 TNFi** | 28 | 23 (82.1) | 5 (17.9) |  |  |  | **1 TNFi** | 25 | 20 (80) | 5 (20) |  |  |  |  |
| **2 TNFis** | 31 | 27 (87.1) | 4 (12.9) |  |  |  | **2 TNFis** | 28 | 22 (78.6) | 6 (21.4) |  |  |  |  |
| **3 or more TNFis** | 31 | 23 (74.2) | 8 (25.8) |  |  |  | **3 or more TNFis** | 25 | 19 (76) | 6 (24) |  |  |  |  |
| **Rheumatoid factor** |  |  |  |  |  |  | **Rheumatoid factor** |  |  |  |  |  |  |  |
| **Negative** | 22 | 18 (81.1) | 4 (18.2) | 0.719 | - | - | **Negative** | 20 | 16 (80) | 4 (20) | 0.831 | - | - |  |
| **Positive** | 83 | 65 (78.3) | 18 (21.7) |  |  |  | **Positive** | 72 | 56 (77.8) | 16 (22.2) |  |  |  |  |
| **ACPAs** |  |  |  |  |  |  | **ACPAs** |  |  |  |  |  |  |  |
| **Negative** | 29 | 26 (89.7) | 3 (10.3) | 0.098 | 2.86 | 0.74-16.43 | **Negative** | 24 | 19 (79.2) | 5 (20.8) | 0.900 | - | - |  |
| **Positive** | 76 | 57 (75) | 19 (25) |  |  |  | **Positive** | 68 | 53 (77.9) | 15 (22.1) |  |  |  |  |
| **DAS28** | 105 | 4.94 ± 1.45 | 4.17 ± 1.34 | 0.032 | 0.69 | 0.49-0.96 | **DAS28** | 92 | 4.8 ± 1.42 | 4.42 ± 1.32 | 0.266 | - | - |  |
| **NPJ** | 105 | 7 (3.5-10) | 6 (1.25-8) | 0.156 | - | - | **NPJ** | 92 | 7 (3-10) | 5 (3-9) | 0.566 | - | - |  |
| **NIJ** | 105 | 3 (1-6) | 2 (0.25-3.75) | 0.191 | - | - | **NIJ** | 92 | 3 (1-5) | 2 (0-6) | 0.629 | - | - |  |
| **PVAS** | 105 | 70 (60-80) | 50 (32.5-60) | 0.006 | 0.96 | 0.94-0.99 | **PVAS** | 92 | 70 (50-80) | 60 (47.5-70) | 0.339 | - | - |  |
| **CRP** | 105 | 2.3 (1.25-4.70) | 2.85 (2-5.13) | 0.483 | - | - | **CRP** | 92 | 2.33 (1.4-5.13) | 2.65 (1.38-3.93) | 0.894 | - | - |  |
| **ESR** | 105 | 22 (10-41.5) | 22 (8.75-32.75) | 0.662 | - | - | **ESR** | 92 | 22 (12-36) | 13.5 (8-39.5) | 0.394 | - | - |  |
| **HAQ** | 105 | 1.75 (1.25-2.06) | 1.25 (0.90-2) | 0.038 | 0.48 | 0.23-0.94 | **HAQ** | 92 | 1.67 ± 0.73 | 1.44 ± 0.73 | 0.216 | - | - |  |
| ***CD80 rs57271503*** |  |  |  |  |  |  | ***CD80 rs57271503*** |  |  |  |  |  |  |  |
| ***AA*** | 2 | 2 (100) | 0 (0) | 0.751 | - | - | ***AA*** | 2 | 2 (100) | 0 (0) | 0.869 | - | - |  |
| ***GG*** | 72 | 58 (80.6) | 14 (19.4) |  |  |  | ***GG*** | 62 | 49 (79) | 13 (21) |  |  |  |  |
| ***AG*** | 31 | 23 (74.2) | 8 (25.8) |  |  |  | ***AG*** | 28 | 21 (75) | 7 (25) |  |  |  |  |
| ***A*** | 33 | 25 (75.8) | 8 (24.2) | 0.575 | - | - | ***A*** | 30 | 23 (76.7) | 7 (23.3) | 0.797 | - | - |  |
| ***G*** | 103 | 81 (78.6) | 22 (21.4) | 0.462 | - | - | ***G*** | 90 | 70 (77.8) | 20 (22.2) | 0.451 | - | - |  |
| ***CD86 rs1129055*** |  |  |  |  |  |  | ***CD86 rs1129055*** |  |  |  |  |  |  |  |
| ***AA*** | 11 | 8 (72.7) | 3 (27.3) | 0.542 | - | - | ***AA*** | 11 | 10 (90.9) | 1 (9.1) | 0.560 | - | - |  |
| ***GG*** | 46 | 35 (76.1) | 11 (23.9) |  |  |  | ***GG*** | 39 | 31 (79.5) | 8 (20.5) |  |  |  |  |
| ***AG*** | 48 | 40 (83.3) | 8 (16.7) |  |  |  | ***AG*** | 42 | 31 (73.8) | 11 (26.2) |  |  |  |  |
| ***A*** | 59 | 48 (81.4) | 11 (18.6) | 0.510 | - | - | ***A*** | 53 | 41 (77.4) | 12 (22.6) | 0.807 | - | - |  |
| ***G*** | 94 | 75 (78.8) | 19 (20.2) | 0.695 | - | - | ***G*** | 81 | 62 (76.5) | 19 (23.5) | 0.445 | - | - |  |
| ***CTLA4 rs3087243*** |  |  |  |  |  |  | ***CTLA4 rs3087243*** |  |  |  |  |  |  |  |
| ***AA*** | 27 | 21 (77.8) | 6 (22.2) | 0.277 | - | - | ***AA*** | 23 | 21 (91.3) | 2 (8.7) | 0.160 | - | - |  |
| ***GG*** | 28 | 25 (89.3) | 3 (10.7) |  |  |  | ***GG*** | 24 | 19 (79.2) | 5 (20.8) |  |  |  |  |
| ***AG*** | 50 | 37 (74.0) | 13 (26.0) |  |  |  | ***AG*** | 45 | 32 (71.1) | 13 (28.9) |  |  |  |  |
| ***A*** | 77 | 58 (75.3) | 19 (24.7) | 0.120 | - | - | ***A*** | 68 | 53 (77.9) | 15 (22.1) | 0.900 | - | - |  |
| ***G*** | 78 | 62 (79.5) | 16 (20.5) | 0.851 | - | - | ***G*** | 69 | 51 (73.9) | 18 (26.1) | 0.079 | 3.66 | 0.76-35.37 |  |
| ***CTLA4 rs5742909*** |  |  |  |  |  |  | ***CTLA4 rs5742909*** |  |  |  |  |  |  |  |
| ***CC*** | 84 | 63 (75) | 21 (25) | 0.122 | - | - | ***CC*** | 75 | 62 (82.7) | 13 (17.3) | 0.055 | - | - |  |
| ***TT*** | 2 | 2 (100) | 0 (0) |  |  |  | ***TT*** | 2 | 2 (100) | 0 (0) |  |  |  |  |
| ***CT*** | 19 | 18 (94.7) | 1 (5.3) |  |  |  | ***CT*** | 15 | 8 (53.3) | 7 (46.7) |  |  |  |  |
| ***C*** | 103 | 81 (78.6) | 22 (21.4) | 0.462 | - | - | ***C*** | 90 | 70 (77.8) | 20 (22.2) | 0.451 | - | - |  |
| ***T*** | 21 | 20 (95.2) | 1 (4.8) | 0.067 | 6.67 | 0.84-52.74 | ***T*** | 17 | 10 (58.8) | 7 (41.2) | 0.048 | 3.28 | 0.89-11.82 |  |
| ***CTLA4 rs231775*** |  |  |  |  |  |  | ***CTLA4 rs231775*** |  |  |  |  |  |  |  |
| ***AA*** | 52 | 41 (78.8) | 11 (21.2) | 0.965 | - | - | ***AA*** | 44 | 39 (88.6) | 5 (11.4) | 0.041 | - | - |  |
| ***GG*** | 6 | 5 (83.3) | 1 (16.7) |  |  |  | ***GG*** | 5 | 4 (80) | 1 (20) |  |  |  |  |
| ***AG*** | 47 | 37 (78.7) | 10 (21.3) |  |  |  | ***AG*** | 43 | 29 (67.4) | 14 (32.6) |  |  |  |  |
| ***A*** | 99 | 78 (78.8) | 21 (21.2) | 0.791 | - | - | ***A*** | 87 | 68 (78.2) | 19 (21.8) | 0.923 | - | - |  |
| ***G*** | 53 | 42 (79.2) | 11 (20.8) | 0.960 | - | - | ***G*** | 48 | 33 (68.8) | 15 (31.2) | 0.021 | 3.49 | 1.06-13.65 |  |
| ABA, abatacept; ACPAs, anti-cyclic citrullinated peptide antibodies; BT, biological therapy; CI_95%_, Confidence interval 95%; CRP, C-reactive protein; csDMARDs, conventional synthetic disease-modifying antirheumatic drugs; DAS28, 28-joints Disease Activity Score; ESR, erythrocyte sedimentation rate; HAQ, Health Assessment Questionnaire score; LDA, Low-disease activity; NIJ, number of inflamed joints; NPJ, number of painful joints; OR, Odds ratio; PVAS, patient’s visual analogue scale; RA, rheumatoid arthritis; TNFi, tumor necrosis factor inhibitor. | | | | | | | | | | | | | |  |
