# Supplementary material for: Impact of Single-Nucleotide Polymorphisms of CTLA-4, CD80 and CD86 on the Effectiveness of Abatacept in Patients with Rheumatoid Arthritis
Source: J Pers Med. 2020 Nov 11;10(4):220. doi: 10.3390/jpm10040220 (PMC7711575; doi:10.3390/jpm10040220)
Supplement: Supplementary file 1 [file jpm-10-00220-s001.zip › Table S18.docx]

**Table S18. Predictors of remission at 6 and 12 months of treatment with abatacept in rheumatoid arthritis patients (bivariate analysis)**

|  | **6 months** | | | | | | | **12 months** | | | | | |  |
| --- | --- | --- | --- | --- | --- | --- | --- | --- | --- | --- | --- | --- | --- | --- |
| **Independent variable** | **Remission** | | | | | | **Independent variable** | **Remission** | | | | | |  |
|  | **N** | **No remission** | **Remission** | **p-value** | **OR** | **CI_95%_** |  | **N** | **No remission** | **Remission** | **p-value** | **OR** | **CI_95%_** |  |
| **Sex** |  |  |  |  |  |  | **Sex** |  |  |  |  |  |  |  |
| **Female** | 76 | 65 (85.5) | 11 (14.5) | 0.240 | - | **-** | **Female** | 64 | 46 (71.9) | 18 (28.1) | 0.466 | - | - |  |
| **Male** | 29 | 22 (75.9) | 7 (24.1) |  |  |  | **Male** | 28 | 18 (64.3) | 10 (35.7) |  |  |  |  |
| **Smoking** |  |  |  |  |  |  | **Smoking** |  |  |  |  |  |  |  |
| **Smokers** | 15 | 10 (66.7) | 5 (33.3) | 0.204 | - | **-** | **Smokers** | 13 | 8 (61.5) | 5 (38.5) | 0.682 | - | - |  |
| **Former-smokers** | 11 | 10 (90.9) | 1 (9.1) |  |  |  | **Former-smokers** | 9 | 6 (66.7) | 3 (33.3) |  |  |  |  |
| **Non-smokers** | 79 | 67 (84.8) | 12 (15.2) |  |  |  | **Non-smokers** | 70 | 50 (71.4) | 20 (28.6) |  |  |  |  |
| **Age at RA diagnosis** | 105 | 45.61 ± 13.41 | 42.83 ± 18.03 | 0.455 | - | **-** | **Age at RA diagnosis** | 92 | 45.66 ± 14.33 | 44.18 ± 15.50 | 0.658 | - | - |  |
| **Disease duration (years)** | 105 | 16 (8.5-22) | 11 (8-17) | 0.180 | - | **-** | **Disease duration (years)** | 92 | 16.5 (10-23.25) | 10 (7-18.50) | 0.039 | 0.94 | 0.89-0.99 |  |
| **Age at ABA start** | 105 | 57.39 ± 12.56 | 51.56 ± 15.34 | 0.087 | 0.97 | **0.93-1.01** | **Age at ABA start** | 92 | 58.11 ± 13.35 | 52.75 ± 13.83 | 0.083 | 0.97 | 0.94-1.00 |  |
| **Duration of ABA (months)** | 105 | 26 (14-43) | 42.5 (28-68.5) | 0.046 | 1.01 | **0.99-1.03** | **Duration of ABA (months)** | 92 | 29 (19-54.75) | 33.5 (24.25 58) | 0.268 | - | - |  |
| **ABA administration** |  |  |  |  |  |  | **ABA administration** |  |  |  |  |  |  |  |
| **Subcutaneous** | 57 | 48 (84.2) | 9 (15.8) | 0.688 | - | **-** | **Subcutaneous** | 50 | 32 (64) | 18 (36) | 0.206 | - | - |  |
| **Intravenous** | 48 | 39 (81.2) | 9 (18.8) |  |  |  | **Intravenous** | 42 | 32 (76.2) | 10 (23.8) |  |  |  |  |
| **Concomitant csDMARDs** |  |  |  |  |  |  | **Concomitant csDMARDs** |  |  |  |  |  |  |  |
| **Methotrexate** | 36 | 30 (83.3) | 6 (16.7) | 0.761 | - | **-** | **Methotrexate** | 32 | 21 (65.6) | 11 (34.4) | 0.402 | - | - |  |
| **Leflunomide** | 14 | 10 (71.4) | 4 (28.6) |  |  |  | **Leflunomide** | 13 | 9 (69.2) | 4 (30.8) |  |  |  |  |
| **Others** | 2 | 2 (100) | 0 (0) |  |  |  | **Others** | 2 | 1 (50) | 1 (50) |  |  |  |  |
| **Concomitant glucocorticoids** |  |  |  |  |  |  | **Concomitant glucocorticoids** |  |  |  |  |  |  |  |
| **Yes** | 89 | 78 (87.6) | 11 (12.4) | 0.002 | 5.52 | **1.71-17.81** | **Yes** | 78 | 56 (71.8) | 22 (28.2) | 0.273 | - | - |  |
| **No** | 16 | 9 (56.2) | 7 (43.8) |  |  |  | **No** | 14 | 8 (57.1) | 6 (42.9) |  |  |  |  |
| **Monotherapy** |  |  |  |  |  |  | **Monotherapy** |  |  |  |  |  |  |  |
| **No** | 99 | 85 (85.9) | 14 (14.1) | 0.001 | 12.14 | **2.03-72.66** | **No** | 86 | 60 (69.8) | 26 (30.2) | 0.873 | - | - |  |
| **Yes** | 6 | 2 (33.3) | 4 (66.7) |  |  |  | **Yes** | 6 | 4 (66.7) | 2 (33.3) |  |  |  |  |
| **Number of previous BTs** | 105 | 2 (1-3) | 1.5 (1-2) | 0.381 | - | **-** | **Number of previous BTs** | 92 | 2 (1-3) | 1 (0-2.25) | 0.081 | 0.68 | 0.44-1.02 |  |
| **Duration of previous BTs (months)** | 105 | 36 (12-60) | 24 (12-36) | 0.157 | - | **-** | **Duration of previous BTs (months)** | 92 | 36 (24-60.25) | 24 (0-36) | 0.008 | 0.98 | 0.97-0.99 |  |
| **Previous BTs** |  |  |  |  |  |  | **Previous BTs** |  |  |  |  |  |  |  |
| **Bionaive** | 15 | 12 (80) | 3 (20) | 0.844 | - | **-** | **Bionaive** | 14 | 5 (35.7) | 9 (64.3) | 0.038 | 5.70 | 1.43-25.78 |  |
| **1 TNFi** | 28 | 22 (78.6) | 6 (21.4) |  |  |  | **1 TNFi** | 25 | 19 (76) | 6 (24) |  |  |  |  |
| **2 TNFis** | 31 | 26 (83.9) | 5 (16.1) |  |  |  | **2 TNFis** | 28 | 22 (78.6) | 6 (21.4) |  |  |  |  |
| **3 or more TNFis** | 31 | 27 (87.1) | 4 (12.9) |  |  |  | **3 or more TNFis** | 25 | 18 (72) | 7 (28) |  |  |  |  |
| **Rheumatoid factor** |  |  |  |  |  |  | **Rheumatoid factor** |  |  |  |  |  |  |  |
| **Negative** | 22 | 18 (81.8) | 4 (18.2) | 0.884 | - | **-** | **Negative** | 20 | 13 (65) | 7 (35) | 0.616 | - | - |  |
| **Positive** | 83 | 69 (83.1) | 14 (16.9) |  |  |  | **Positive** | 72 | 51 (70.8) | 21 (29.2) |  |  |  |  |
| **ACPAs** |  |  |  |  |  |  | **ACPAs** |  |  |  |  |  |  |  |
| **Negative** | 29 | 25 (86.2) | 4 (13.8) | 0.573 | - | **-** | **Negative** | 24 | 17 (70.8) | 7 (29.2) | 0.875 | - | - |  |
| **Positive** | 76 | 62 (81.6) | 14 (18.4) |  |  |  | **Positive** | 68 | 47 (69.1) | 21 (30.9) |  |  |  |  |
| **DAS28** | 105 | 5.05 ± 1.27 | 3.43 ± 1.57 | <0.001 | 0.42 | **0.25-0.63** | **DAS28** | 92 | 4.93 ± 1.19 | 4.23 ± 1.73 | 0.060 | 0.69 | 0.49-0.96 |  |
| **NPJ** | 105 | 8 (4.5-11) | 3 (2-5.75) | <0.001 | 0.75 | **0.62-0.87** | **NPJ** | 92 | 8 (4-11) | 5 (2-8) | 0.012 | 0.89 | 0.80-0.99 |  |
| **NIJ** | 105 | 3 (1-6) | 0 (0-1) | <0.001 | 0.64 | **0.45-0.83** | **NIJ** | 92 | 3 (1.75-6) | 1 (0-4) | 0.042 | 0.89 | 0.75-1.02 |  |
| **PVAS** | 105 | 70 (60-80) | 30 (20-57.5) | <0.001 | 0.94 | **0.91-0.97** | **PVAS** | 92 | 70 (60-80) | 50 (30-70) | <0.001 | 0.96 | 0.93-0.98 |  |
| **CRP** | 105 | 2.60 (1.43-5.25) | 2.05 (1.03-4.30) | 0.618 | - | **-** | **CRP** | 92 | 2.65 (1.45-5.13) | 2.04 (1.25-4.55) | 0.462 | - | - |  |
| **ESR** | 105 | 27 (13-42.5) | 11 (6.25-18) | <0.001 | 0.94 | **0.89-0.98** | **ESR** | 92 | 21.5 (9.75-44.25) | 22 (11.75-32.50) | 0.665 | - | - |  |
| **HAQ** | 105 | 1.75 (1.5-2) | 0.80 (0.5-1.2) | <0.001 | 0.25 | **0.10-0.55** | **HAQ** | 92 | 1.78 ± 0.69 | 1.25 ± 0.69 | 0.001 | 0.33 | 0.16-0.65 |  |
| ***CD80 rs57271503*** |  |  |  |  |  |  | ***CD80 rs57271503*** |  |  |  |  |  |  |  |
| ***AA*** | 2 | 1 (50) | 1 (50) | 0.459 | - | **-** | ***AA*** | 2 | 2 (100) | 0 (0) | 0.601 | - | - |  |
| ***GG*** | 72 | 60 (83.3) | 12 (16.7) |  |  |  | ***GG*** | 62 | 42 (67.7) | 20 (32.3) |  |  |  |  |
| ***AG*** | 31 | 26 (83.9) | 5 (16.1) |  |  |  | ***AG*** | 28 | 20 (71.4) | 8 (28.6) |  |  |  |  |
| ***A*** | 33 | 27 (81.8) | 6 (18.2) | 0.848 | - | **-** | ***A*** | 30 | 22 (73.3) | 8 (26.7) |  |  |  |  |
| ***G*** | 103 | 86 (83.5) | 17 (16.5) | 0.315 | - | **-** | ***G*** | 90 | 62 (68.9) | 28 (31.1) |  |  |  |  |
| ***CD86 rs1129055*** |  |  |  |  |  |  | ***CD86 rs1129055*** |  |  |  |  |  |  |  |
| ***AA*** | 11 | 8 (72.7) | 3 (27.3) | 0.425 | - | **-** | ***AA*** | 11 | 7 (63.6) | 4 (36.4) | 0.225 | - | - |  |
| ***GG*** | 46 | 37 (80.4) | 9 (19.6) |  |  |  | ***GG*** | 39 | 24 (61.5) | 15 (38.5) |  |  |  |  |
| ***AG*** | 48 | 42 (87.5) | 6 (12.5) |  |  |  | ***AG*** | 42 | 33 (78.6) | 9 (21.4) |  |  |  |  |
| ***A*** | 59 | 50 (84.7) | 9 (15.3) | 0.561 | - | **-** | ***A*** | 53 | 40 (75.5) | 13 (24.5) | 0.151 | - | - |  |
| ***G*** | 94 | 79 (84) | 15 (16) | 0.396 | - | **-** | ***G*** | 81 | 57 (70.4) | 24 (29.6) | 0.730 | - | - |  |
| ***CTLA4 rs3087243*** |  |  |  |  |  |  | ***CTLA4 rs3087243*** |  |  |  |  |  |  |  |
| ***AA*** | 27 | 27 (100) | 0 (0) | 0.022 | - | **-** | ***AA*** | 23 | 19 (82.6) | 4 (17.4) | 0.127 | - | - |  |
| ***GG*** | 28 | 21 (75) | 7 (25) |  |  |  | ***GG*** | 24 | 18 (75) | 6 (25) |  |  |  |  |
| ***AG*** | 50 | 39 (78) | 11 (22) |  |  |  | ***AG*** | 45 | 27 (60) | 18 (40) |  |  |  |  |
| ***A*** | 77 | 66 (85.7) | 11 (14.3) | 0.243 | - | **-** | ***A*** | 68 | 46 (67.6) | 22 (32.4) | 0.501 | - | - |  |
| ***G*** | 78 | 60 (76.9) | 18 (23.1) | 0.006 | - | **-** | ***G*** | 69 | 45 (65.2) | 24 (34.8) | 0.117 | - | - |  |
| ***CTLA4 rs5742909*** |  |  |  |  |  |  | ***CTLA4 rs5742909*** |  |  |  |  |  |  |  |
| ***CC*** | 84 | 71 (84.5) | 13 (15.5) | 0.388 | - | **-** | ***CC*** | 75 | 53 (70.7) | 22 (29.3) | 0.457 | - | - |  |
| ***TT*** | 2 | 1 (50) | 1 (50) |  |  |  | ***TT*** | 2 | 2 (100) | 0 (0) |  |  |  |  |
| ***CT*** | 19 | 15 (78.9) | 4 (21.1) |  |  |  | ***CT*** | 15 | 9 (60) | 6 (40) |  |  |  |  |
| ***C*** | 103 | 86 (83.5) | 17 (16.5) | 0.315 | - | **-** | ***C*** | 91 | 62 (68.9) | 28 (31.1) | 1 | - | - |  |
| ***T*** | 21 | 16 (76.2) | 5 (23.8) | 0.349 | - | **-** | ***T*** | 17 | 11 (64.7) | 6 (35.3) | 0.629 | - | - |  |
| ***CTLA4 rs231775*** |  |  |  |  |  |  | ***CTLA4 rs231775*** |  |  |  |  |  |  |  |
| ***AA*** | 52 | 47 (90.4) | 5 (9.6) | 0.110 | - | **-** | ***AA*** | 44 | 32 (72.7) | 12 (27.3) | 0.772 | - | - |  |
| ***GG*** | 6 | 5 (83.3) | 1 (16.7) |  |  |  | ***GG*** | 5 | 3 (60) | 2 (40) |  |  |  |  |
| ***AG*** | 47 | 35 (74.5) | 12 (25.5) |  |  |  | ***AG*** | 43 | 29 (67.4) | 14 (32.6) |  |  |  |  |
| ***A*** | 99 | 82 (82.8) | 17 (17.2) | 1 | - | **-** | ***A*** | 87 | 61 (70.1) | 26 (29.9) | 0.638 | - | - |  |
| ***G*** | 53 | 40 (75.5) | 13 (24.5) | 0.043 | 3.06 | 1.05-10.20 | ***G*** | 48 | 32 (66.7) | 16 (33.3) | 0.528 | - | - |  |
| ABA, abatacept; ACPAs, anti-cyclic citrullinated peptide antibodies; BT, biological therapy; CI_95%_, Confidence interval 95%; CRP, C-reactive protein; csDMARDs, conventional synthetic disease-modifying antirheumatic drugs; DAS28, 28-joints Disease Activity Score; ESR, erythrocyte sedimentation rate; HAQ, Health Assessment Questionnaire score; NIJ, number of inflamed joints; NPJ, number of painful joints; OR, Odds ratio; PVAS, patient’s visual analogue scale; RA, rheumatoid arthritis; TNFi, tumor necrosis factor inhibitor. | | | | | | | | | | | | | |  |
